# Psychiatric traits and intracerebral hemorrhage: A Mendelian randomization study

**DOI:** 10.3389/fpsyt.2022.1049432

**Published:** 2023-01-04

**Authors:** Qingduo Wang, Yajie Qi, Yuping Li, Zhengcun Yan, Xiaodong Wang, Qiang Ma, Can Tang, Xiaoguang Liu, Min Wei, Hengzhu Zhang

**Affiliations:** ^1^Department of Neurosurgery, The Yangzhou School of Clinical Medicine of Dalian Medical University, Dalian, China; ^2^Department of Neurosurgery, Clinical Medical College, Yangzhou University, Yangzhou, China

**Keywords:** Mendelian randomization, intracerebral hemorrhage, psychiatric traits, genome-wide association study, causality

## Abstract

**Background:**

Psychiatric traits have been associated with intracerebral hemorrhage (ICH) in observational studies, although their causal relationships remain uncertain. We used Mendelian randomization analyses to infer causality between psychiatric traits and ICH.

**Methods:**

We collected data from genome-wide association studies of ICH (*n* = 361,194) and eight psychiatric traits among Europeans, including mood swings (*n* = 451,619), major depressive disorder (*n* = 480,359), attention-deficit/hyperactivity disorder (*n* = 53,293), anxiety (*n* = 459,560), insomnia (*n* = 462,341), schizophrenia (*n* = 77,096), neuroticism (*n* = 374,323), and bipolar disorder (*n* = 51,710). We performed a series of bidirectional two-sample Mendelian randomization and related sensitivity analyses. A Bonferroni corrected threshold of *p* < 0.00625 (0.05/8) was considered to be significant, and *p* < 0.05 was considered suggestive of evidence for a potential association.

**Results:**

Mendelian randomization analyses revealed suggestive positive causality of mood swings on ICH (odds ratio = 1.006, 95% confidence interval = 1.001–1.012, *p* = 0.046), and the result was consistent after sensitivity analysis. However, major depressive disorder (*p* = 0.415), attention-deficit/hyperactivity disorder (*p* = 0.456), anxiety (*p* = 0.664), insomnia (*p* = 0.699), schizophrenia (*p* = 0.799), neuroticism (*p* = 0.140), and bipolar disorder (*p* = 0.443) are not significantly associated with the incidence of ICH. In the reverse Mendelian randomization analyses, no causal effects of ICH on mood swings (*p* = 0.565), major depressive disorder (*p* = 0.630), attention-deficit/hyperactivity disorder (*p* = 0.346), anxiety (*p* = 0.266), insomnia (*p* = 0.102), schizophrenia (*p* = 0.463), neuroticism (*p* = 0.261), or bipolar disorder (*p* = 0.985) were found.

**Conclusion:**

Our study revealed that mood swings are suggestively causal of ICH and increase the risk of ICH. These results suggest the clinical significance of controlling mood swings for ICH prevention.

## 1. Introduction

Intracerebral hemorrhage (ICH) is the most lethal form of stroke and is commonly caused by hypertension, vascular malformation, tumor (1), trauma (2), cerebral amyloid angiopathy (3), intracranial aneurysms (4), and oral anticoagulants (5). ICH accounts for approximately 10–15% of all stroke types among Europeans (6) with high disability and fatality rates (7), thereby increasing the burden on patients, medical staff, and society. Psychiatric traits usually coexist in patients with ICH. Several observational studies have revealed that ICH is associated with psychiatric traits, including mood swings (8), major depressive disorder (MDD) (9–11), attention-deficit/hyperactivity disorder (ADHD) (12), anxiety (13), insomnia (14), schizophrenia (15, 16), neuroticism (17), and bipolar disorder (BPD) (18). Depression, mood swings, anxiety, and cognitive function disorders are more common in patients with ICH compared to those with acute ischemic stroke (10, 11, 13). In addition, previous research reported that approximately 20% of ICH survivors suffered from depressive mood which adversely affected their quality of life (9). Previous studies provided strong evidence for an association between psychiatric traits and ICH, and our study aimed to explore the causality between psychiatric traits and ICH. Potential residual confounding or reverse causality bias in observational studies has limited the ability to identify causal relationships (19). Therefore, better evaluation methods are needed to prove causality between psychiatric traits and ICH.

With the development of Mendelian randomization (MR) and genome-wide association studies (GWASs), causality between exposure factors and disease outcomes can be identified (20). MR is a method used to infer the relationships between exposure factors and outcomes based on the principle of independent assortment of alleles during meiosis, as they are independent of behavioral and environmental factors that confound epidemiological studies (21). MR assesses the effect of exposure factors on outcomes using genetic variations as instrumental variables (IVs), thereby avoiding potential biases in observational studies (22). A recent MR study indicated that MDD increased the risk of ischemic stroke (23), but the relationships between ICH and psychiatric traits are still unclear.

Here, we collected GWAS data to infer and genetically investigate the causality between ICH and the eight psychiatric traits by bidirectional two-sample MR analyses. Our research may provide evidence to prevent and treat psychiatric traits in patients with ICH.

## 2. Materials and methods

### 2.1. Hypotheses of the MR study

**FIGURE 1:**
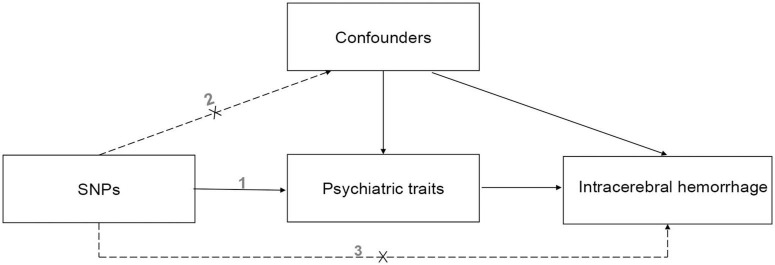
The causal directed acyclic graph of the Mendelian randomization study. The design hypotheses are that the genetic variants are associated with psychiatric traits, but not with confounders, and the genetic variants are associated with the risk of ICH only through psychiatric traits. And similar hypotheses are applicable for the reverse MR analysis. SNP, single nucleotide polymorphism.

The three hypotheses of our MR study are that (i) genetic IVs are strongly associated with the eight psychiatric traits such as anxiety and depression, (ii) they are not associated with any potential confounders, and (iii) they do not affect ICH independently of the eight psychiatric traits. A causal directed acyclic graph is shown in Figure 1. And similar hypotheses are applicable for the reverse MR analysis.

### 2.2. Data extraction

We collected summary data for ICH and the eight psychiatric traits from published studies with European population samples (Table 1). The definitions of ICH and the 8 psychiatric traits are displayed in Supplementary Table 1. We selected a large GWAS (1,253 cases and 359,941 controls) of ICH and download data from.^1^ We were able to download data of mood swings (204,412 cases and 247,207 controls), anxiety (158,565 cases and 300,995 controls), insomnia (total sample size = 462,341), and neuroticism (total sample size = 374,323) from the MRC Integrative Epidemiology Unit at the University of Bristol.^2^ There were 44 independent significant loci for MDD (24) from 135,458 cases to 344,901 controls. The sample size of the ADHD study (25) was 55,374 and included 96% European data; there were 12 independent loci and we selected only the European population sample including 19,099 cases and 34,194 controls. The schizophrenia study (26) included 33,640 cases and 43,456 controls with 128 independent associations. The sample size for the BPD study (27) was 51,710 including 20,352 cases and 31,358 controls with 30 loci, including 20 new loci.^3^

**TABLE 1:** Details from GWAS data.

Psychiatric traits	Total sample size	Cases, *n*	Controls, *n*	Population	Consortium
ICH	361,194	1,253	359,941	European	Neale lab
MDD	480,359	135,458	344,901	European	PGC
ADHD	53,293	19,099	34,194	European	PGC
Mood swings	451,619	204,412	247,207	European	MRC-IEU
Anxiety	459,560	158,565	300,995	European	MRC-IEU
Insomnia	462,341	462,341*	462,341*	European	MRC-IEU
Schizophrenia	77,096	33,640	43,456	European	SCZ-PGC
Neuroticism	374,323	374,323*	374,323*	European	MRC-IEU
BPD	51,710	20,352	31,358	European	BPD-PGC

BPD-PGC, bipolar disorder working group of the psychiatric genomics consortium; MRC-IEU, the MRC integrative epidemiology unit at the university of bristol; PGC, psychiatric genomics consortium; SCZ-PGC, schizophrenia working group of the psychiatric genomics consortium; ADHD, attention deficit/hyperactivity disorder; BPD, bipolar disorder; GWAS, genome-wide association study; ICH, intracerebral hemorrhage; MDD, major depressive disorder.

*Number of the total sample size.

### 2.3. IV selection and bidirectional MR analyses

**FIGURE 2:**
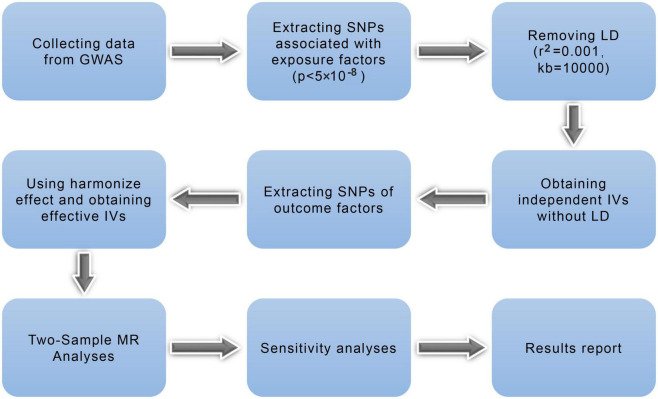
Mendelian randomization study flowchart. The blue boxes represent research steps while gray arrows indicate the overall direction. GWAS, genome-wide association study; IV, instrumental variable; LD, linkage disequilibrium; MR, Mendelian randomization; SNP, single-nucleotide polymorphism.

Figure 2 illustrates the research workflow. Based on the required MR, valid IVs were selected from the collected data. First, we selected single-nucleotide polymorphisms (SNPs) associated with exposure factors (*p* < 5 × 10^–8^) as candidate IVs. Second, linkage disequilibrium was removed for the screened SNPs, leaving independent IVs without linkage disequilibrium (*r*^2^ = 0.001, kb = 10,000) (28). Third, the potentially related phenotypes were detected using the online database “PhenoScanner^4^” by filtration of *r*^2^ > 0.8 and *p* < 5 × 10^–8^. Fourth, we extracted information regarding SNPs and outcomes; in this step, no proxy SNPs were used. Fifth, we used the harmonization effect to align the effect alleles of the IVs and ensured that the SNP effects on exposures and outcomes were consistent with the effect alleles. Valid IVs of the SNPs associated with ICH as outcomes and eight psychiatric traits as exposure factors (group A) were obtained using the above process and then used for two-sample MR and sensitivity analyses. In the reverse MR analysis, we repeated the above four steps and obtained valid IVs associated with the eight psychiatric traits as outcomes and ICH as exposure factors (group B). Then, we calculated F statistics for each valid IV in both groups to exclude the weak IV bias by using the following formula (29):

$$F=\frac{R^{2}\,\left(n-1-k\right)}{\left(1-R^{2}\right)\,k}$$

where *n*, k, and R^2^, are sample size, number of IVs, and the variance explained by IVs, respectively. A previous study suggested that a weak IV is considered when the F statistic is less than 10, and an *F-*value greater than 11 may ensure that relative bias will be <10% at least 95% of the time, regardless of the number of IVs used in the analysis (29–31). Then, we set the *p*-value < 0.00625 (0.05/8) was considered to be significant according to the Bonferroni multiple correction criterion. A *p*-value < 0.05 but above the Bonferroni-corrected threshold is considered suggestive evidence of a potential causal relationship.

### 2.4. Statistical and sensitivity analyses

All statistical analyses were performed using the “Two Sample MR,” “Mendelian Randomization,” and “MR-PRESSO” (Mendelian Randomization Pleiotropy RESidual Sum and Outlier) packages in R (version 4.2.1; The R Foundation for Statistical Computing, Vienna, Austria). Valid IVs were used to perform two-sample MR analyses. The inverse variance weighted method was used in the primary analyses; the weighted median, simple mode, weight mode, and MR-Egger methods were used in the secondary analyses. The sensitivity analysis included heterogeneity, pleiotropy, and leave-one-out sensitivity tests.

We tested for heterogeneity using the inverse variance weighted method and MR-Egger regression, with results revealed by the *p*-value of Cochran’s *Q*-test. The intercept term of the MR-Egger method indicated horizontal pleiotropy (32). MR-PRESSO was used to detect and remove horizontal pleiotropy (33) by removing the outlier-corrected horizontal pleiotropy (*p* < 0.05 for detecting outliers) (34) and testing the difference in estimates before and after outlier correction (35). In addition, we performed a leave-one-out sensitivity test (36) to assess the robustness of the MR results. A *p*-value less than 0.05 was considered statistically significant in the pleiotropy and heterogeneity tests.

## 3. Results

### 3.1. MR and sensitivity analyses results in group A

**FIGURE 3:**
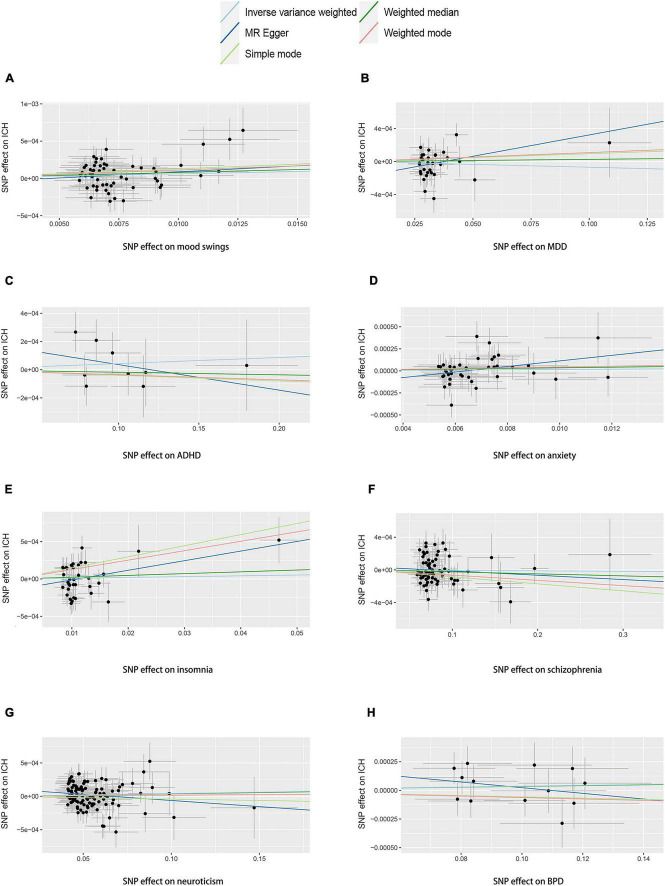
Scatter plots of group A. Scatter plots of the eight MR results from the eight psychiatric traits related to ICH. Each point in the scatter plot represents an IV. The line on each point reflects the 95% CI, and the horizontal coordinate is the effect of SNPs on **(A)** mood swings, **(B)** MDD, **(C)** ADHD, **(D)** anxiety, **(E)** insomnia, **(F)** schizophrenia, **(G)** neuroticism, and **(H)** BPD. The vertical coordinate is the effect of SNPs on ICH. SNP effects were plotted into lines for the inverse-variance weighted test (light blue line), MR-Egger regression (dark blue line), simple mode (light green line), weighted median (dark green line), and weighted mode (pink line). The slope of the line corresponds to the causal estimation. A positive slope indicates that mood swings had a positive effect on ICH. ADHD, attention deficit/hyperactivity disorder; BPD, bipolar disorder; CI, confidence interval; ICH, intracerebral hemorrhage; IV, instrumental variable; MDD, major depressive disorder; MR, Mendelian randomization; SNP, single-nucleotide polymorphism.

**FIGURE 4:**
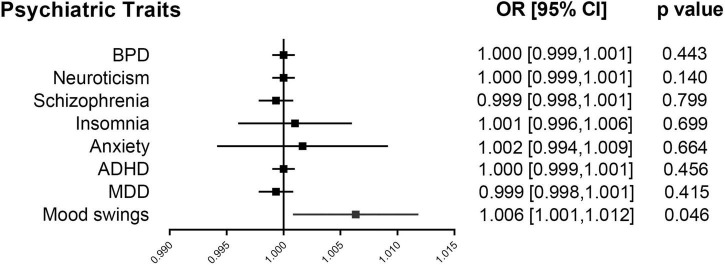
Forest plot of group A. Mendelian randomization associations for eight psychiatric traits on ICH. Results derived from IVW analyses. ADHD, attention deficit/hyperactivity disorder; BPD, bipolar disorder; CI, confidence interval; ICH, intracerebral hemorrhage; IVW, inverse variance weighted; MDD, major depressive disorder; OR, odds ratio.

We performed two-sample MR analyses on the eight groups of data in group A. After obtained for significant SNPs (*p* < 5 × 10^–8^), the linkage disequilibrium was removed, and then we found 17 SNPs of psychiatric traits associated with phenotypes such as smoking, hypertension and alcohol consumption. The information on effective IVs are shown in Supplementary Tables 2–9 after removed the above 17 SNPs and the removed SNPs are shown in Supplementary Table 10. These valid IVs were used to complete the MR study and obtain the results. The scatter plots are shown in Figure 3. Each point in the scatter plot represents an IV and the line on each point reflects the 95% confidence interval. The horizontal coordinate is the effect of SNPs on exposure factors (psychiatric traits), the vertical coordinate is the effect of SNPs on outcomes (ICH), and the colored lines indicate the MR fit results indicating that mood swings exhibit a suggestive risk effect on ICH, with an odds ratio of 1.006 (95% confidence interval = 1.001–1.012, *p* = 0.046), meaning that the risk of ICH increases by 1.006 per unit increase in log-odds of mood swings. However, no evidence demonstrated that MDD (*p* = 0.415), ADHD (*p* = 0.456), anxiety (*p* = 0.664), insomnia (*p* = 0.699), schizophrenia (*p* = 0.799), neuroticism (*p* = 0.140), or BPD (*p* = 0.443) are causally associated with ICH. The F statistics for each IV in Group A are shown in Supplementary Tables 2–9. No evidence suggested weak IV bias in group A (each F statistics >29). We then summarized the results of group A into a forest plot (Figure 4).

**FIGURE 5:**
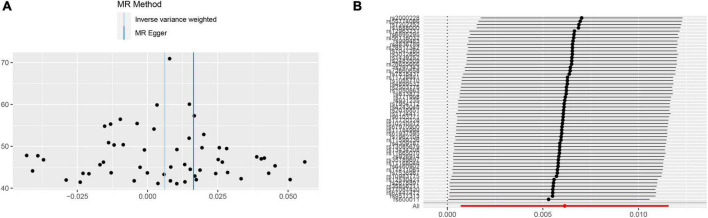
Sensitivity analyses for mood swings on ICH group. **(A)** The funnel plot of the causality of mood swings on ICH was symmetrically distributed overall, suggesting that no significant heterogeneity exists. **(B)** The leave-one-out graph indicated that all lines were located to the right of the “0 line,” indicating that the removal of any SNPs had no fundamental effect on the results, suggesting that the MR results were reliable. ICH, intracerebral hemorrhage; MR, Mendelian randomization; SNP, single nucleotide polymorphism.

In the sensitivity analyses, Cochran’s *Q*-test revealed no heterogeneity (*p* = 0.131) in the mood swings-ICH subgroup, and the funnel plot (Figure 5A) of the causality of mood swings and ICH was symmetrically distributed overall. No horizontal pleiotropy (*p* = 0.550) was observed in the mood swings-ICH subgroup. In the leave-one-out graph (Figure 5B), all lines are located to the right of the “0 line,” indicating that the removal of any SNPs had no fundamental effect on the results, suggesting that the MR results were robust and reliable in this group. Furthermore, Cochran’s *Q*-test revealed no heterogeneity (*p* > 0.05) or horizontal pleiotropy (*p* > 0.05) in the other seven subgroups of group A. The leave-one-out analysis showed that no specific SNP affected the overall estimate (Supplementary Figures 1–7).

### 3.2. MR and sensitivity analyses results in group B

In the reverse MR analyses, the number of relevant SNPs (Supplementary Tables 11–18) obtained for ICH as exposure factors was lower in group B. We decreased the criteria for selecting IVs (*p* < 5 × 10^–6^), and no evidence suggested weak IV bias existed in group B (each F statistics >21). The results indicate that mood swings (*p* = 0.565), MDD (*p* = 0.630), ADHD (*p* = 0.346), anxiety (*p* = 0.266), insomnia (*p* = 0.102), schizophrenia (*p* = 0.463), neuroticism (*p* = 0.261), and BPD (*p* = 0.985) were not significantly associated with the incidence of ICH and no causal effects of ICH on the eight psychiatric traits were revealed. Scatter plots of group B are shown in Supplementary Figures 8–15. In addition, Cochran’s *Q*-test revealed no heterogeneity (*p* > 0.05) or horizontal pleiotropy (*p* > 0.05) in group B. No specific SNPs affected the MR estimate in the leave-one-out analysis (Supplementary Figures 16–23).

## 4. Discussion

In this study, we first explored the causality of ICH and eight psychiatric traits *via* bidirectional two-sample MR analyses by leveraging GWAS summary data. Our study provides suggestive evidence that genetically increasing odds of mood swings are causally associated with the risk of ICH, whereas the other seven psychiatric traits had no significant genetic effect on ICH. In the reverse MR analyses, no evidence supported the presence of a causal relationship between the genetically determined risk of ICH and psychiatric traits.

Our MR results provide genetic evidence of a causal relationship between mood swings and ICH risk. By using data from GWASs, we showed that genetically associated mood swings are associated with a higher risk of ICH, suggesting that preventive strategies focusing on mood stability will reduce the risk of ICH. A meta-analysis suggested a higher incidence and mortality from cardiocerebral vascular disease in people with severe mental illness than in those without (37). Some mental illnesses are associated with insulin resistance (38, 39); patients with severe mental illness are more likely to develop diabetes with more severe and frequent microvascular and macrovascular complications, and insulin resistance may further contribute to hypertension, thereby increasing the risk of cardiocerebral vascular disease (40). In addition, a recent MR study suggested that mood swings are related to cardiocerebral vascular disease risk (41). Evidence from prospective studies implies that mood swings are correlated with blood pressure, which may increase the incidence of ICH (42). Our findings on the effect of mood swings on ICH are compatible with known clinical research.

However, the pathophysiological mechanisms linking mood swings to ICH development are unclear, and several current studies have attempted to explain their mechanisms. Observational studies have shown that oxidative stress is associated with psychiatric traits (43). Total bilirubin is an important antioxidant that exhibits antioxidant, anti-inflammatory, and cytoprotective properties during oxidative stress (44). In terms of oxidative stress, mood swings may decrease total bilirubin levels (45), and lower total bilirubin levels are generally associated with higher blood pressure (44) which increases the risk of ICH from ruptured blood vessels. Inflammatory factors also play an important role in psychiatric traits (46): mood swings may increase the levels of interleukin-6 (47) and cyclooxygenase-2 (48) which may increase the ICH risk and severity, such as larger ICH volumes and perihematomal edema volumes. In addition, alterations in microglia (49, 50), white matter degeneration (51, 52), and aberrant monoaminergic neuron development (53, 54) may also play a role in the relationship between mood swings and ICH. Therefore, further studies are still needed to elucidate these complex mechanisms between mood swings and ICH.

Several clinical studies indicated that ICH was associated with psychiatric traits, including insomnia (14, 55) and schizophrenia (15). ADHD in a small proportion of children may be associated with grade 3 or 4 intraventricular hemorrhage (12). In addition, MDD was reported to share genetic risk factors with cerebrovascular disease and may share common pathophysiological mechanisms, thus explaining the relationship between MDD and ICH (56). On the other hand, whether there is reverse causality between psychiatric traits and ICH has been previously debated. Clinical research demonstrated that greater disability and worse quality of life may increase the risk of depression (10). MDD after ICH may reflect acute mechanical disruption of neural pathways and regions responsible for cognitive and affective function, which is thought to be associated with larger hematoma size or intraventricular hemorrhage (57). ICH patients also often exhibit anxiety that persists for many years after onset (58). Furthermore, cerebral amyloid angiopathy-related ICH displayed prolonged depression and higher resistance to antidepressant treatment (59). Although previous clinical research suggested that psychiatric traits were associated with ICH, in our MR research, there were no genetic causal relationships for the other seven psychiatric traits on the development of ICH, including insomnia, anxiety, MDD, BPD, ADHD, neuroticism, and schizophrenia, and no genetic evidence proved that ICH causes any of these traits. This may be due to several possible reasons. The first is that the discrepancy may be related to other residual confounding bias in observational studies, including blood pressure, diabetes, and microvascular complications (37), which limit the ability to identify causal relationships. The second possible reason is that the number of valid IVs for each trait is small in the reverse MR study, and weak IV bias should be considered, which needs more relevant data to improve further statistics. The third possible reason is that the psychiatric traits after ICH may be associated with physical disability, reducing quality of life, and decreasing social identity in patients with ICH. Therefore, we should not ignore the psychiatric traits associated with ICH, and early intervention and therapy for these traits and adequate social support are beneficial to improve prognosis in patients with ICH.

This study had some limitations. First, our sample was based on a European population. This restriction of our analyses to the European population minimized bias by demographics, but also limits the generalizability of our results for non-European populations. Follow-up studies are required to confirm our results in different ethnicities. Second, psychiatric traits vary widely (60); we only selected eight psychiatric traits in this study, and other psychiatric traits should be included in further studies. Third, the present study did not separately analyze the causality between psychiatric traits and ICH subtypes, such as ventricular, parenchymal, and mixed hemorrhage, which should be considered in follow-up studies. Fourth, psychiatric traits may have some interaction; further research is needed after adjusting for other traits. Our results in our two-sample MR study can only explain to a limited extent the causality between psychiatric traits and ICH. Fifth, as a limitation of the two-sample MR method, sample overlap may exist in our study that may increase weak IV bias. We are temporarily unable to verify or improve this limitation due to data limitations. However, large F statistics of IVs may minimize this dilemma (61).

## 5. Conclusion

In conclusion, we performed bidirectional two-sample MR analyses after collecting GWAS data and concluded that there is suggestive evidence that mood swings potentially cause ICH. Severe mood swings increase the incidence of ICH. The other seven psychiatric traits (MDD, ADHD, insomnia, schizophrenia neuroticism, anxiety, and BPD) had no causality with ICH. In the reverse MR analyses, no genetic evidence implicated ICH as the cause of mood swings, MDD, schizophrenia, ADHD, BPD, insomnia, neuroticism, or anxiety. These results suggest the clinical significance of controlling mood swings for ICH prevention.

## Data availability statement

The original contributions presented in this study are included in the article/Supplementary material, further inquiries can be directed to the corresponding author.

## Author contributions

QW and YQ: concept and design and drafting of the manuscript. QW, XW, and ZY: acquisition, analysis, and interpretation of data. YL and HZ: critical revision of the manuscript for important intellectual content. QW, CT, and QM: statistical analysis. YQ, QW, and XL: charting. HZ: obtained funding and supervision. MW: administrative and technical support. All authors contributed to the article and approved the submitted version.

## Footnotes

1
http://www.nealelab.is/uk-biobank/

2
https://gwas.mrcieu.ac.uk/

3
http://www.med.unc.edu/pgc/

4
www.phenoscanner.medschl.cam.ac.uk/phenoscanner
