# Supplementary material for: Psychiatric traits and intracerebral hemorrhage: A Mendelian randomization study
Source: Front Psychiatry. 2023 Jan 4;13:1049432. doi: 10.3389/fpsyt.2022.1049432 (PMC9850495; doi:10.3389/fpsyt.2022.1049432)
Supplement: Supplementary file 1 [file Data_Sheet_1.docx]

| **Index for supplementary Figure S1-S23** |  |
| --- | --- |
| [**Figure S1.** Leave-one-out graph for major depressive disorder on ICH](#S1) | Page 1 |
| [**Figure S2.** Leave-one-out graph for attention deficit/hyperactivity disorder on ICH](#S2) | Page 2 |
| [**Figure S3.** Leave-one-out graph for anxiety on ICH](#S3) | Page 3 |
| [**Figure S4.** Leave-one-out graph for insomnia on ICH](#S4) | Page 4 |
| [**Figure S5.** Leave-one-out graph for schizophrenia on ICH](#S5) | Page 5 |
| [**Figure S6.** Leave-one-out graph for neuroticism on ICH](#S6) | Page 6 |
| [**Figure S7.** Leave-one-out graph for bipolar disorder on ICH](#S7) | Page 7 |
| [**Figure S8.** Scatter plot for ICH on mood swings](#S8) | Page 8 |
| [**Figure S9.** Scatter plot for ICH on major depressive disorder](#S9) | Page 9 |
| [**Figure S10.** Scatter plot for ICH on attention deficit/hyperactivity disorder](#S10) | Page 10 |
| [**Figure S11.** Scatter plot for ICH on anxiety](#S11) | Page 11 |
| [**Figure S12.** Scatter plot for ICH on insomnia](#S12) | Page 12 |
| [**Figure S13.** Scatter plot for ICH on schizophrenia](#S13) | Page 13 |
| [**Figure S14.** Scatter plot for ICH on neuroticism](#S14) | Page 14 |
| [**Figure S15.** Scatter plot for ICH on bipolar disorder](#S15) | Page 15 |
| [**Figure S16.** Leave-one-out graph for ICH on mood swings](#S16) | Page 16 |
| [**Figure S17.** Leave-one-out graph for ICH on major depressive disorder](#S17) | Page 17 |
| [**Figure S18.** Leave-one-out graph for ICH on attention deficit/hyperactivity disorder](#S18) | Page 18 |
| [**Figure S19.** Leave-one-out graph for ICH on anxiety](#S19) | Page 19 |
| [**Figure S20.** Leave-one-out graph for ICH on insomnia](#S20) | Page 20 |
| [**Figure S21.** Leave-one-out graph for ICH on schizophrenia](#S21) | Page 21 |
| [**Figure S22.** Leave-one-out graph for ICH on neuroticism](#S22) | Page 22 |
| [**Figure S23.** Leave-one-out graph for ICH on bipolar disorder](#S23) | Page 23 |

**
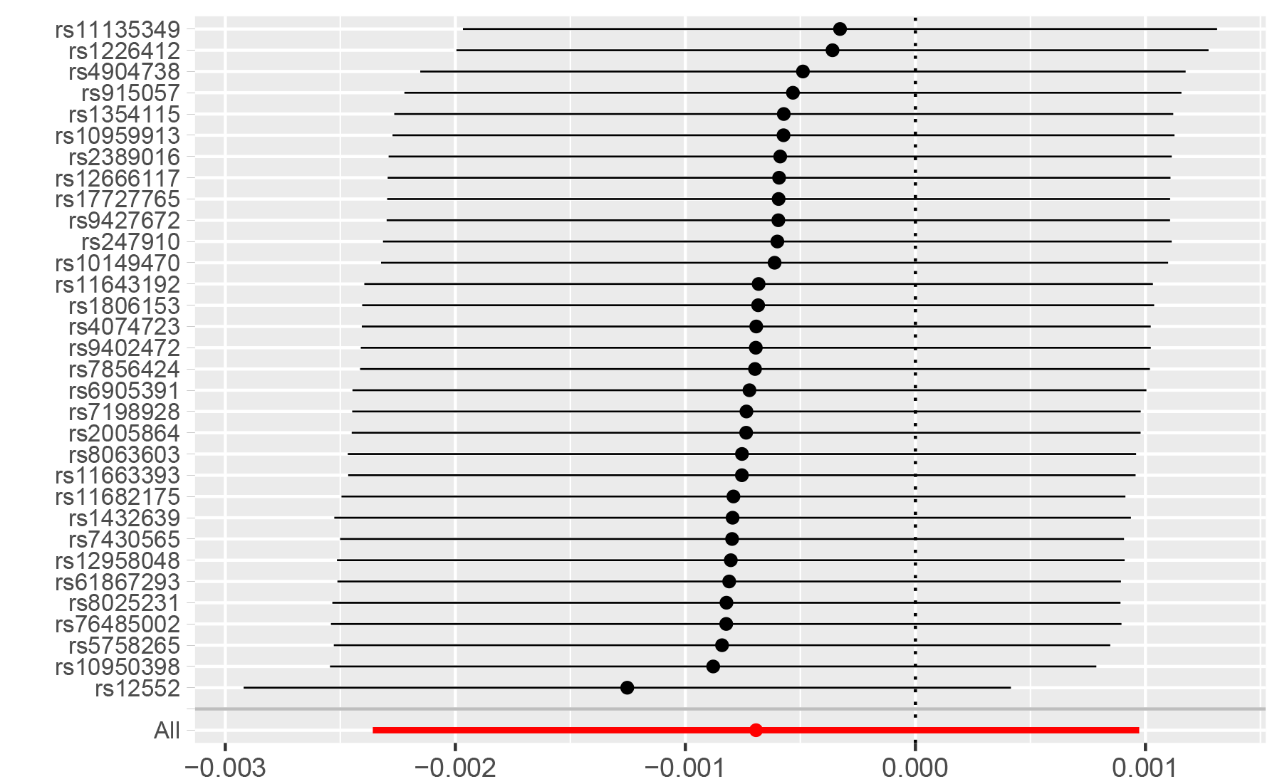
Figure S1. Leave-one-out graph for major depressive disorder on ICH**


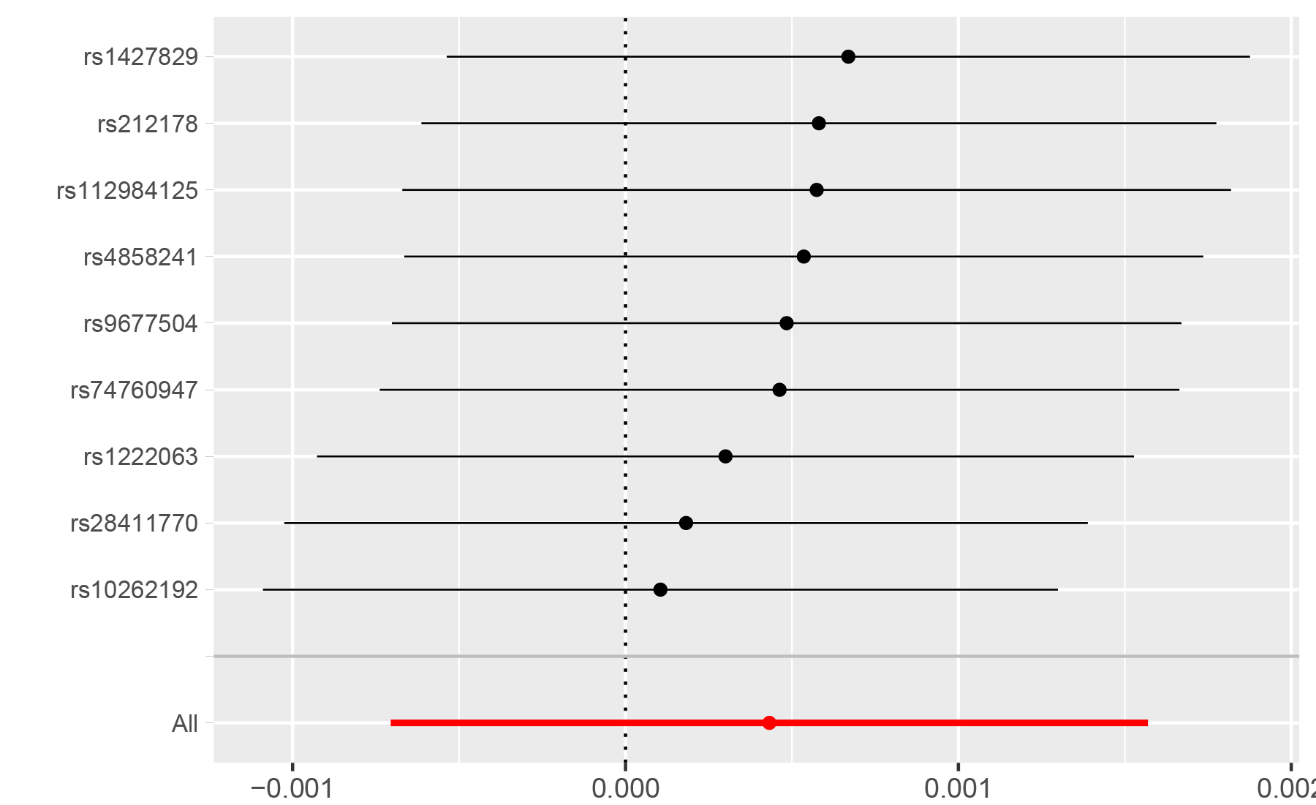
**Figure S2. Leave-one-out graph for attention deficit/hyperactivity disorder on ICH**


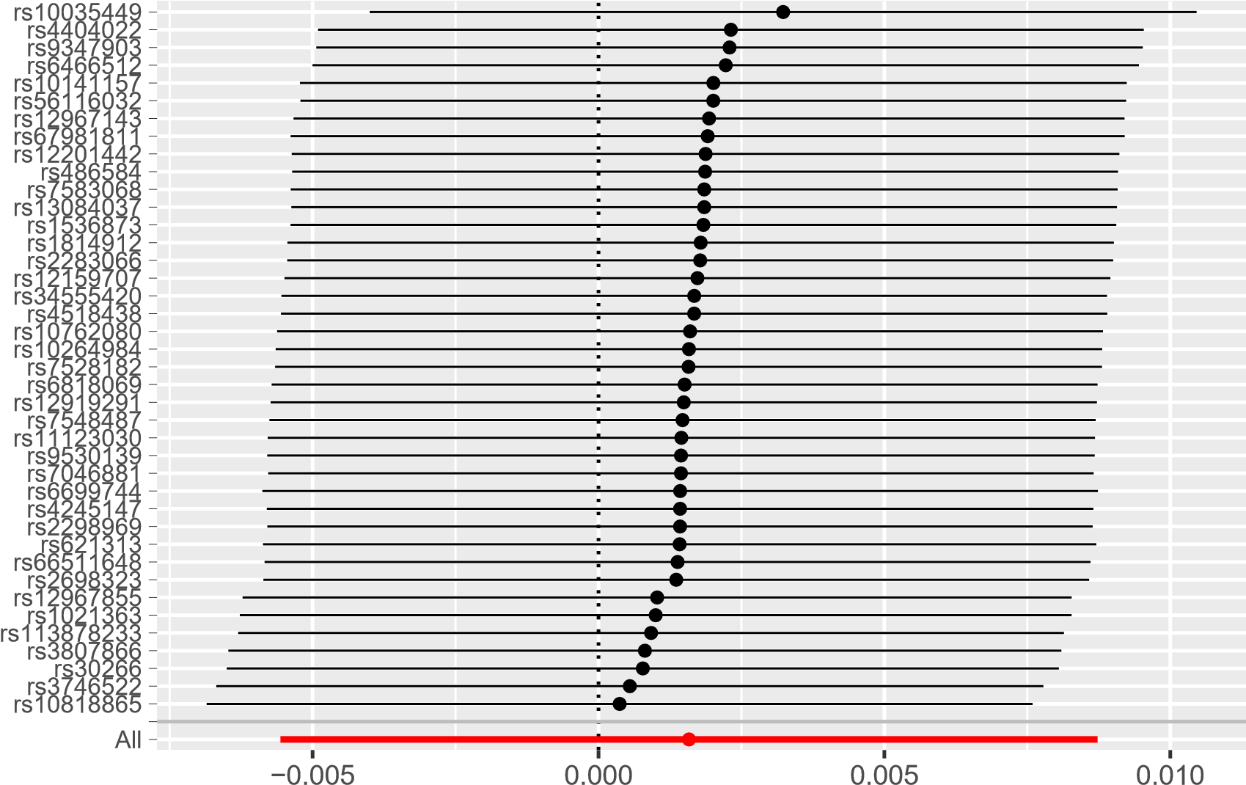
**Figure S3. Leave-one-out graph for anxiety on ICH**


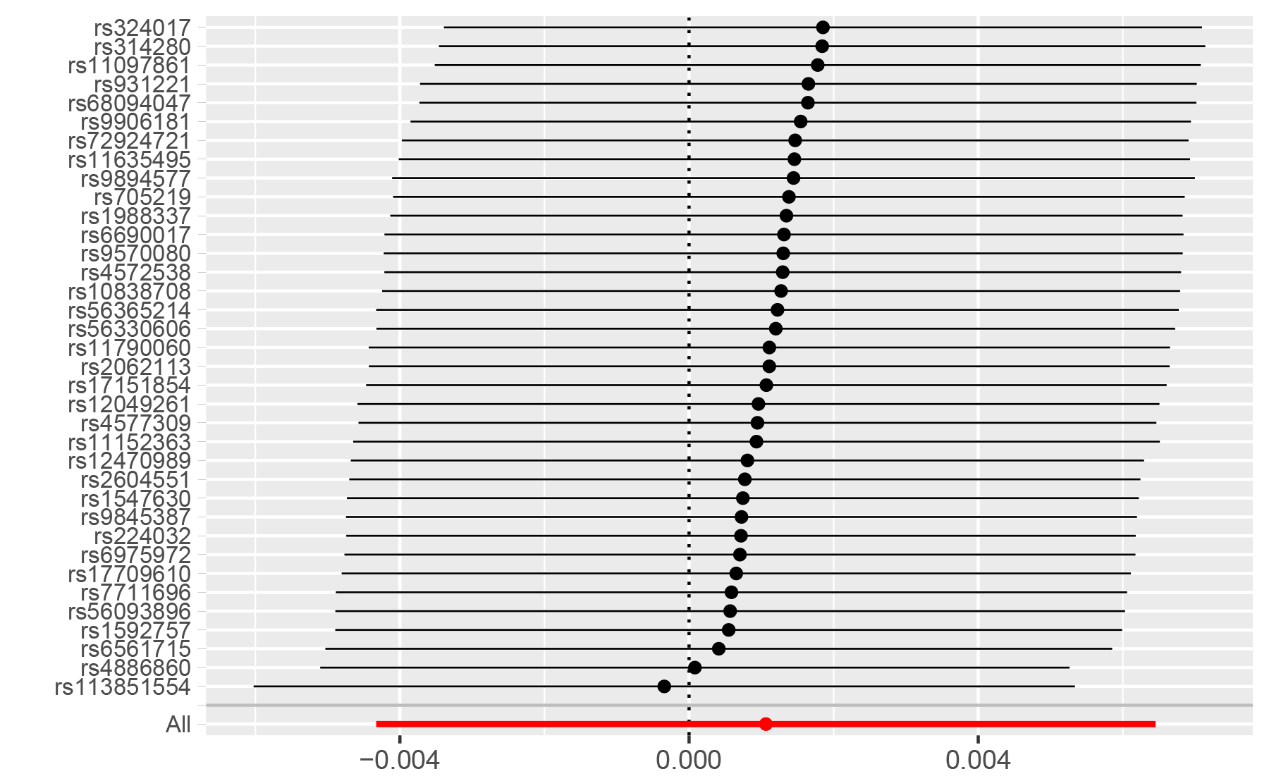
**Figure S4. Leave-one-out graph for insomnia on IC****H**

**
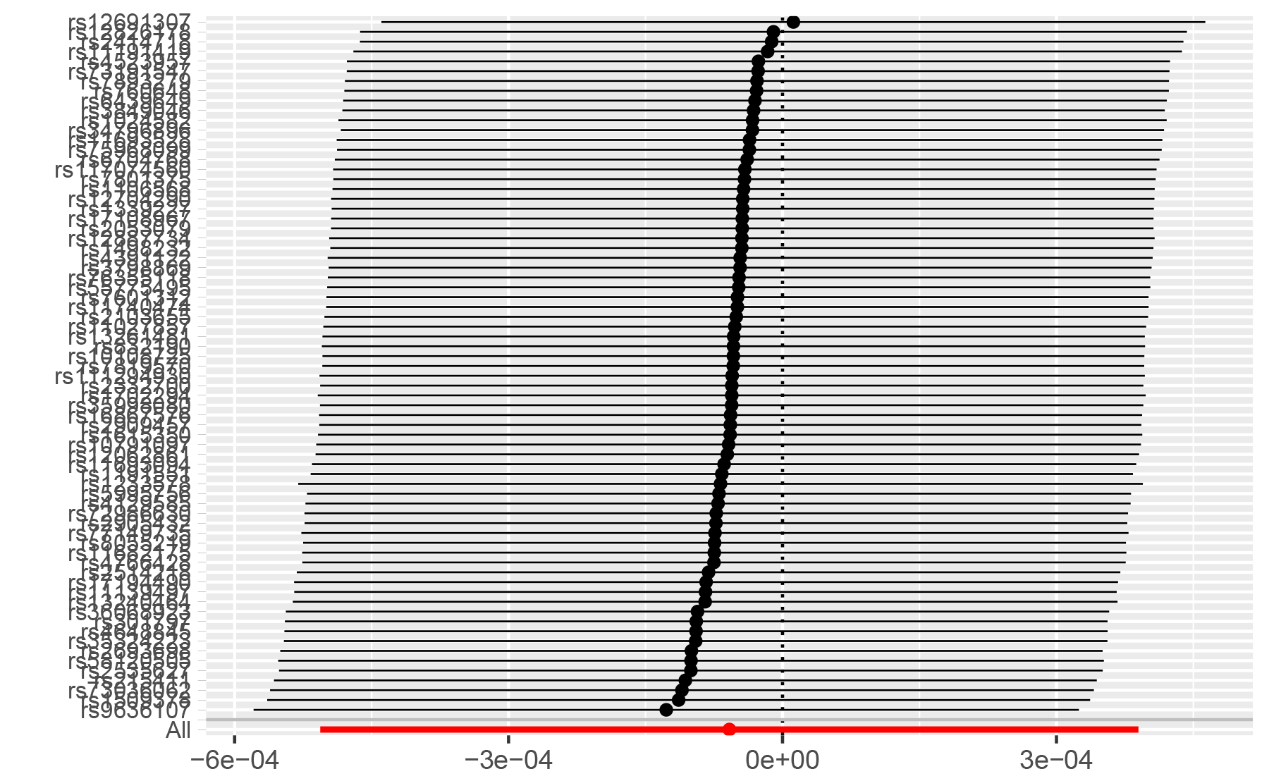
Figure S5. Leave-one-out graph for schizophrenia o****n ICH**

**
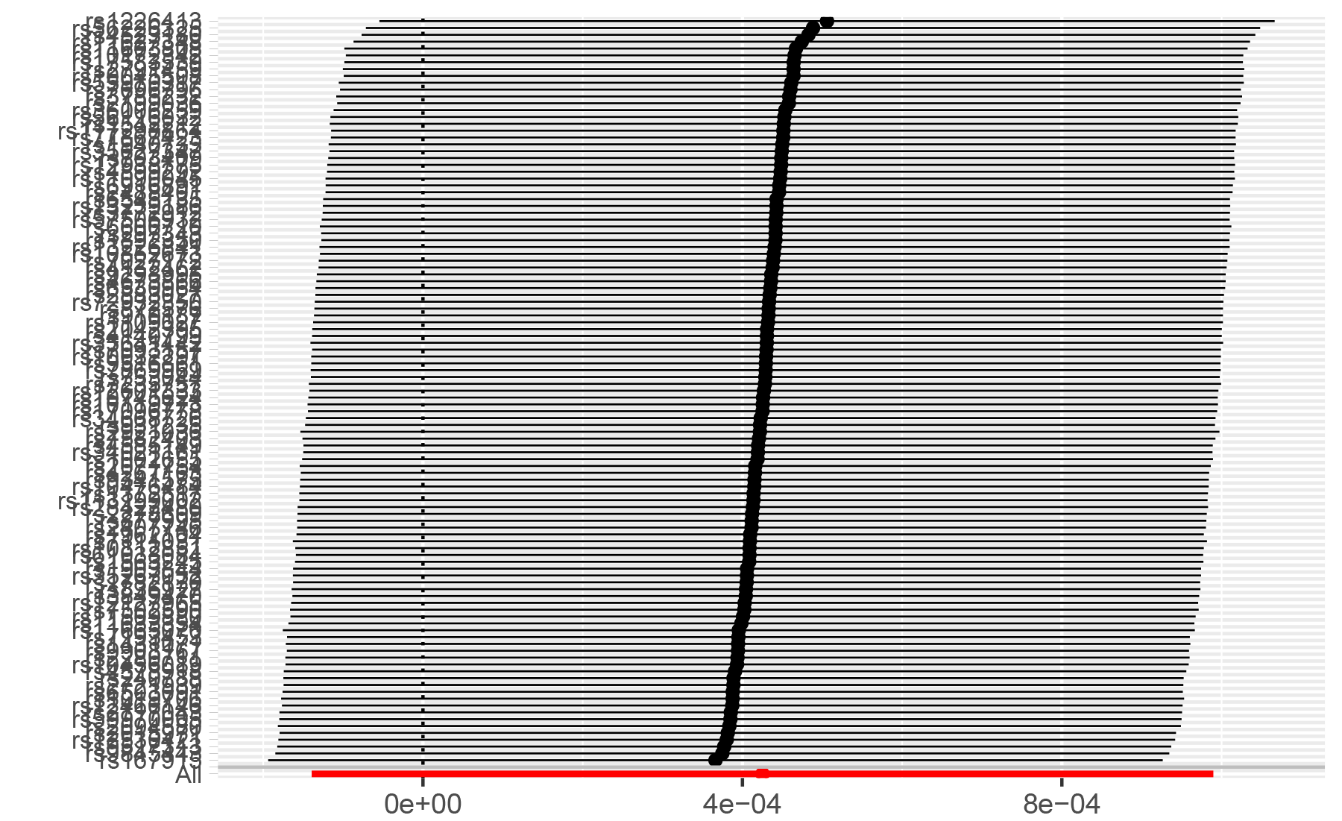
Figure S6. Leave-one-out graph for neuroticism on** **ICH**

**
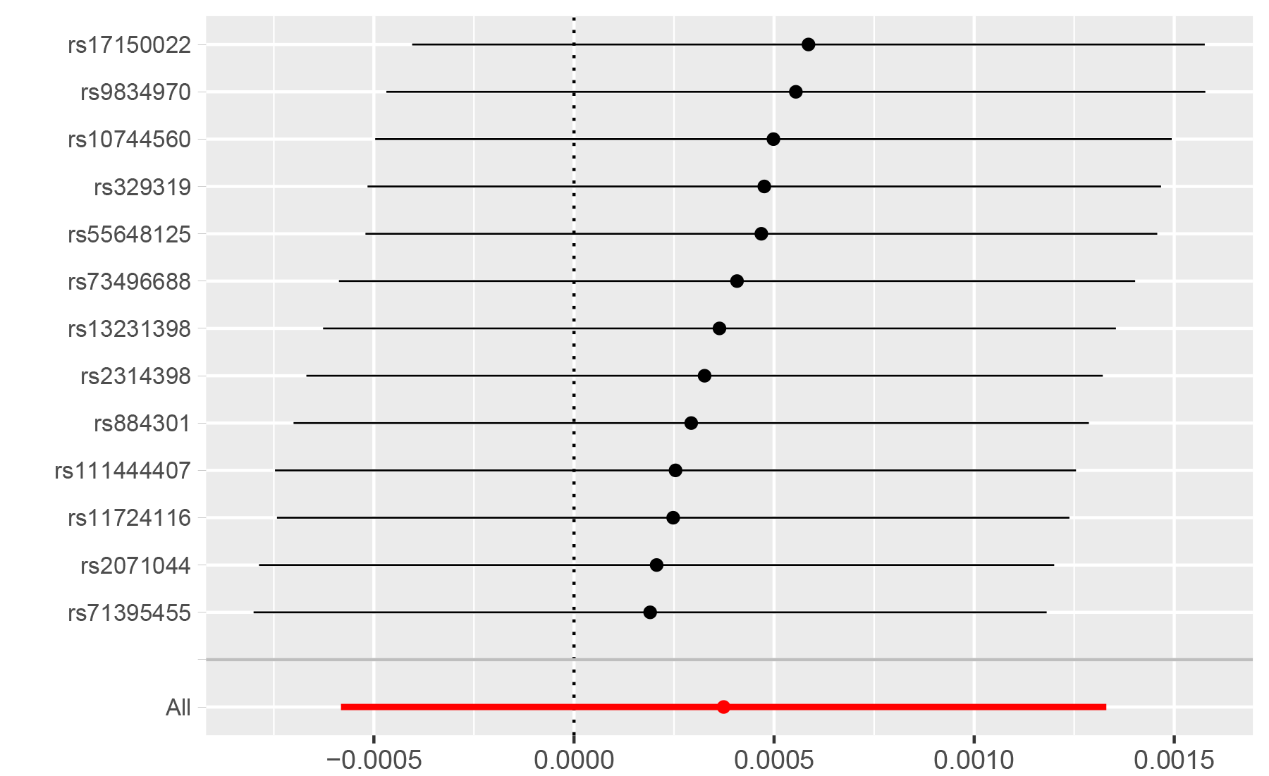
Figure S7. Leave-one-out graph for bipolar disorde****r on ICH**


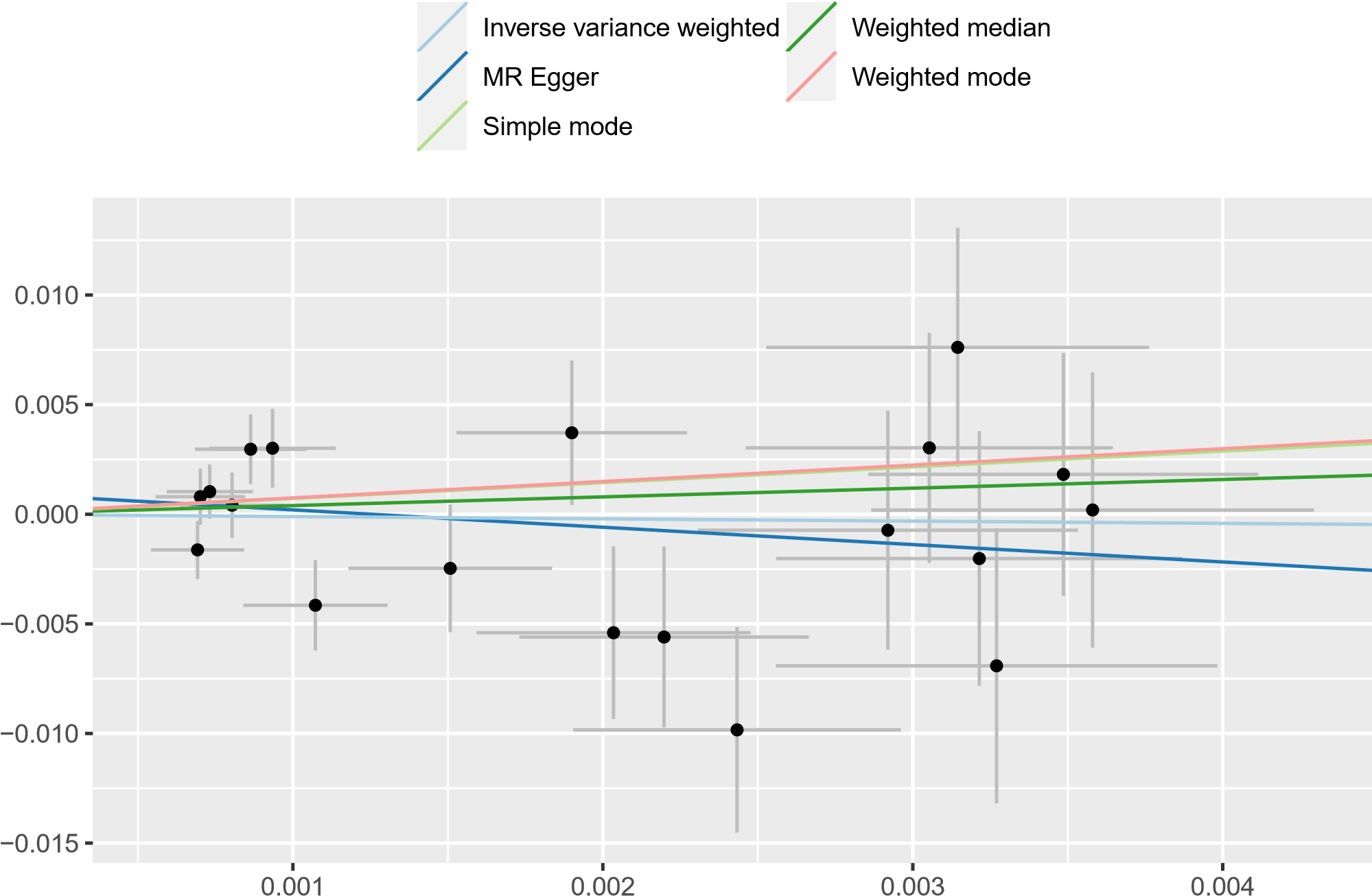
**Figure S8. Scatter plot for ICH on mood swings**

**Figure S9. Scatter plot for ICH on major depres****sive disorder**

−6e−04

−3e−04

0e+00

3e−04

6e−04

0.0007

0.0009

0.0011

0.0013

Inverse variance weighted

MR Egger

Simple mode

Weighted median

Weighted mode

oses − secondary ICD10: F32.9 Depressive episode, unspecified || id:ukb−b

**Figure S10. Scatter plot for ICH on attention de****ficit/hyperactivity disorder**

−0.10

−0.05

0.00

0.05

0.0005

0.0010

0.0015

0.0020

Inverse variance weighted

MR Egger

Simple mode

Weighted median

Weighted mode


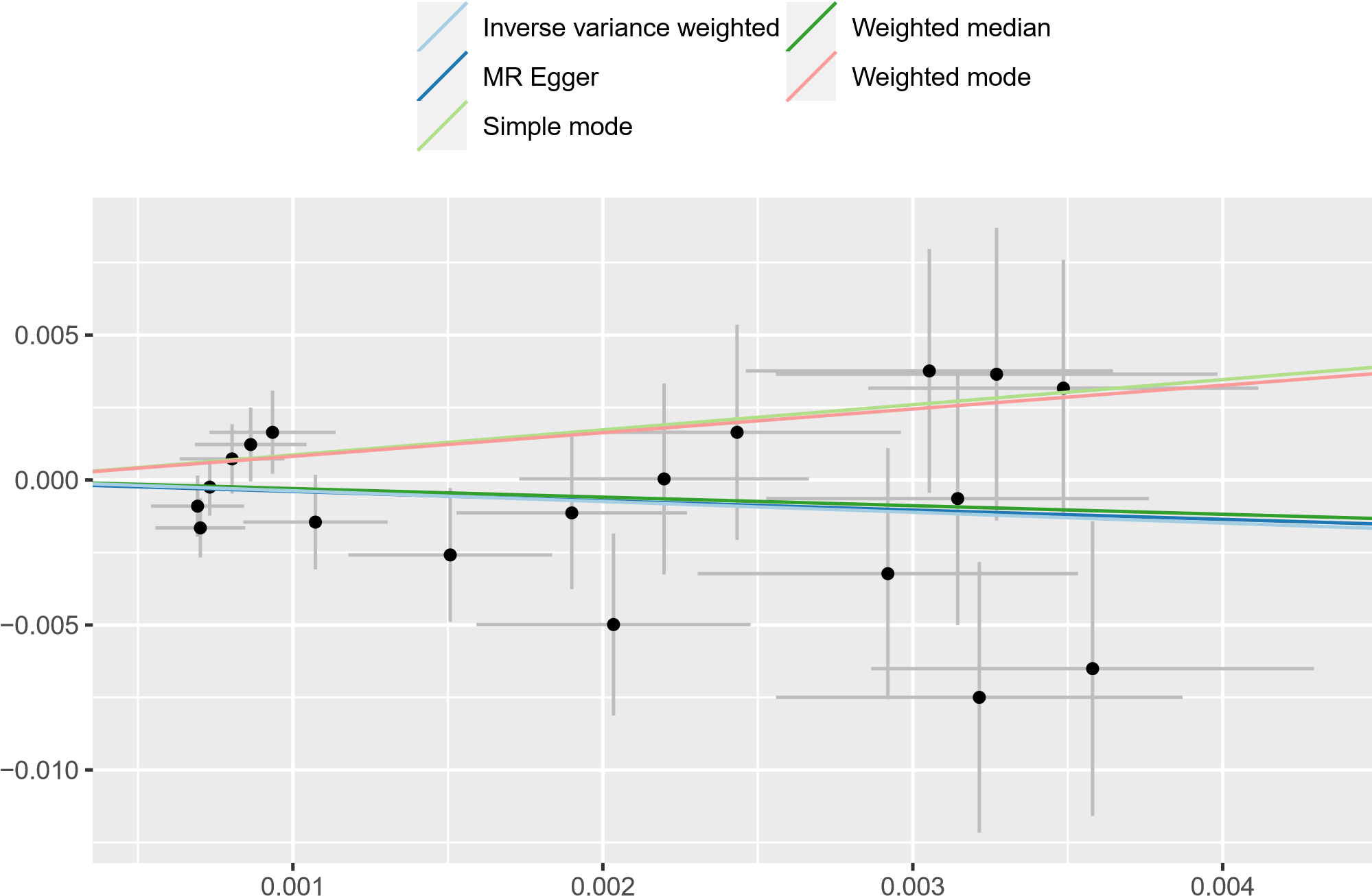
**Figure S11. Scatter plot for ICH on anxiety**

**Figure S12. Scatter plot for ICH on insomnia**

**
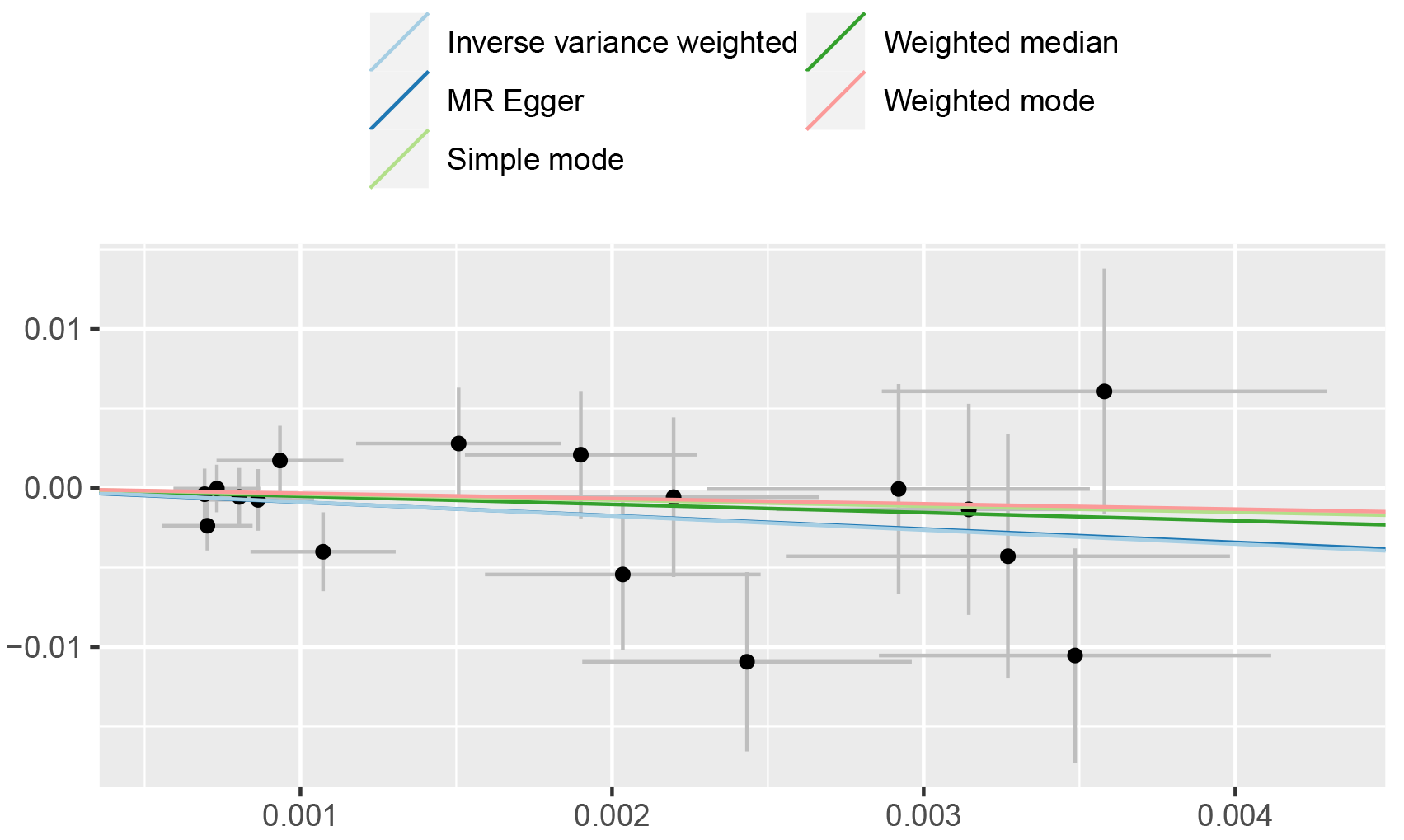
**

**Figure S13. Scatter plot for ICH on schizophren****ia**


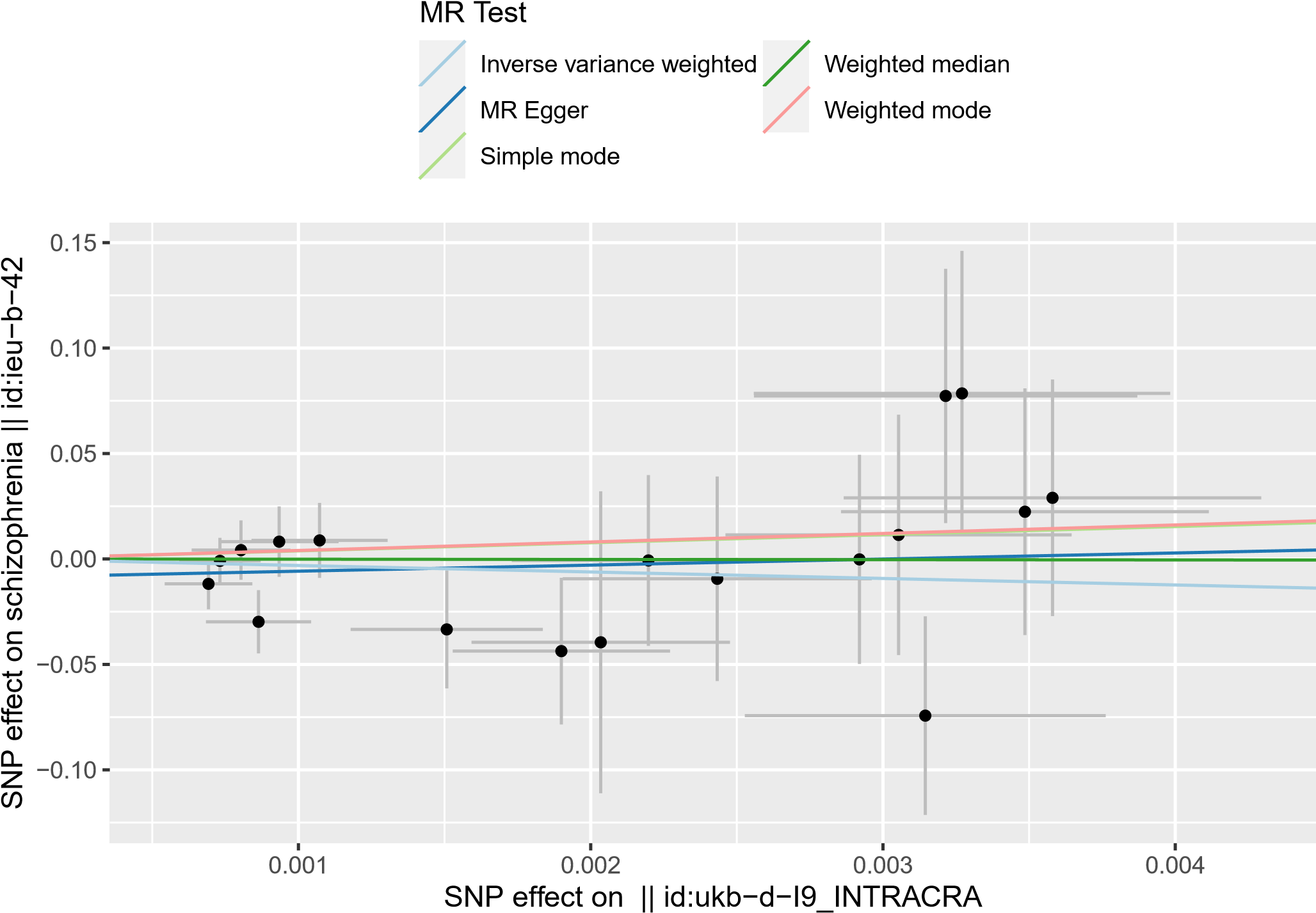


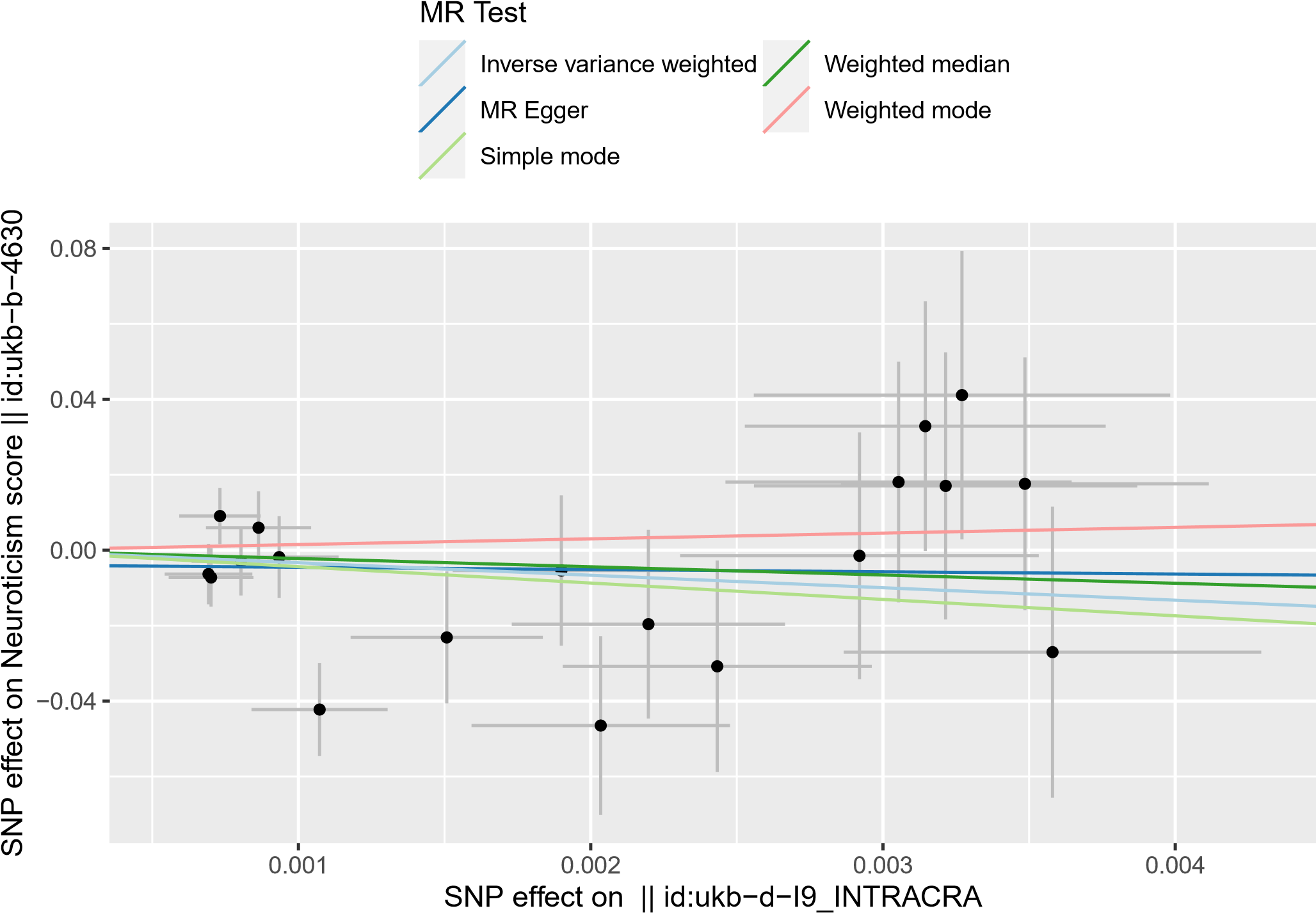
**Figure S14. Scatter plot for ICH on neuroticism**


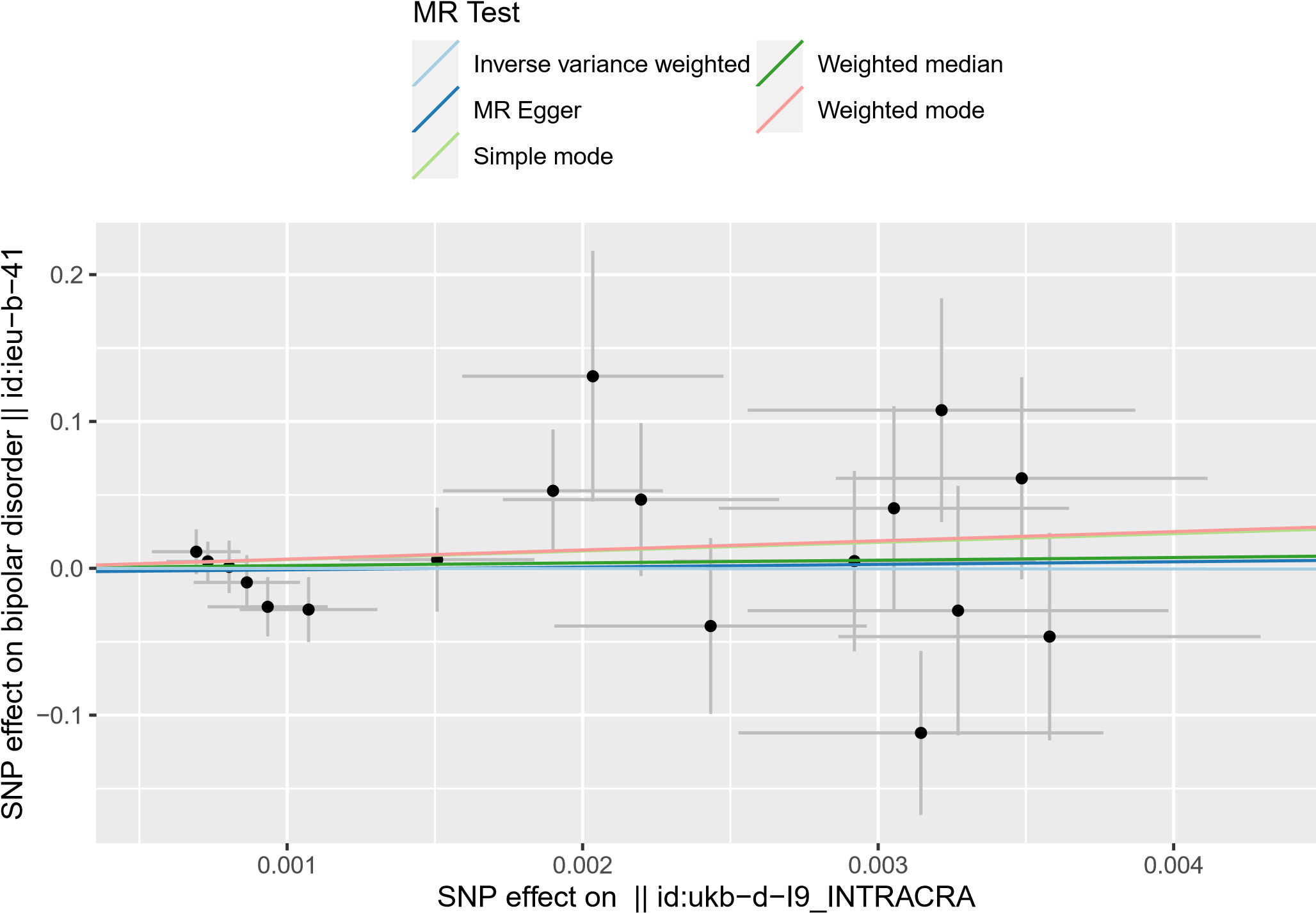
**Figure S15. Scatter plot for ICH on bipolar diso****rder**


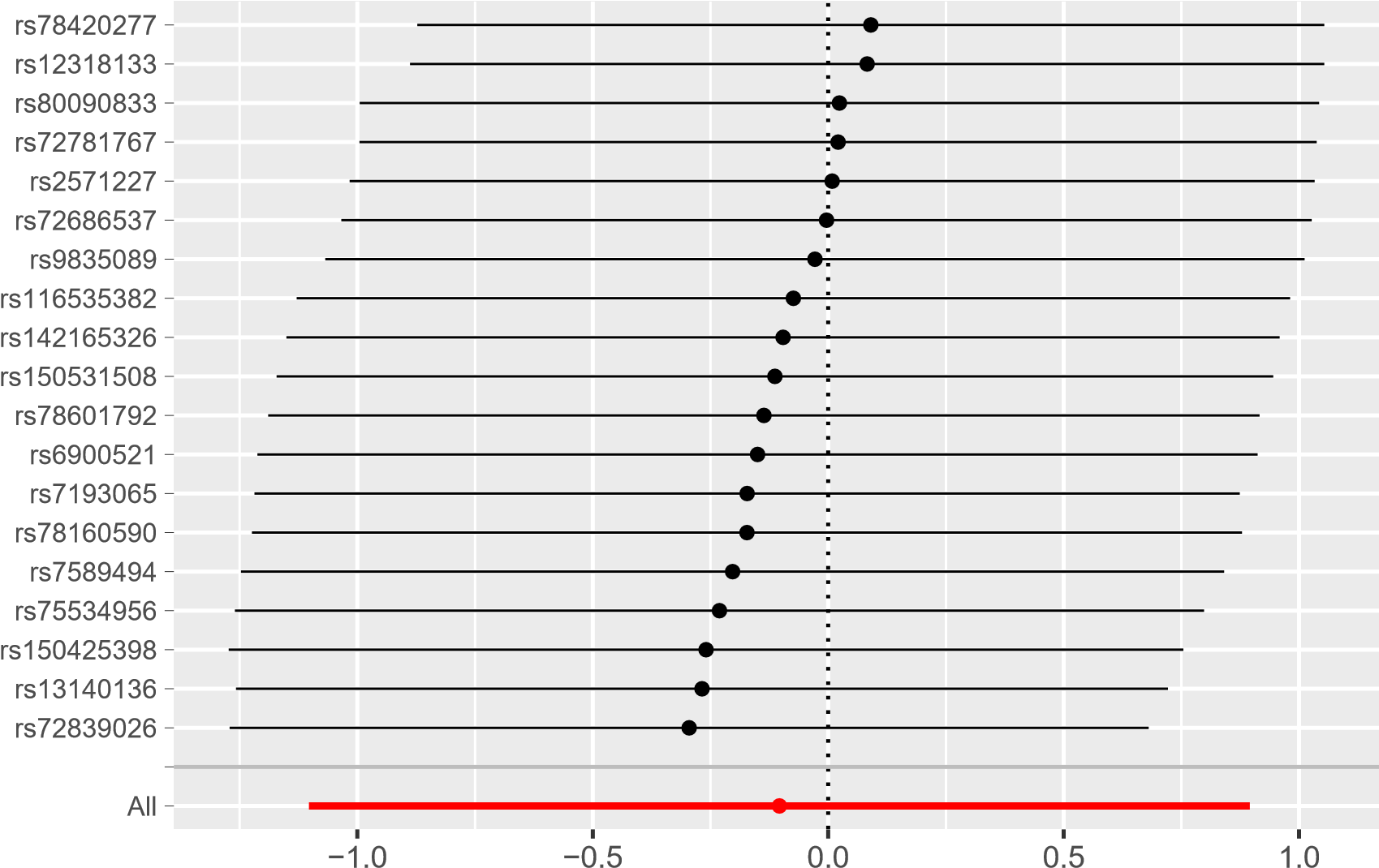
**Figure S16. Leave-one-out graph for ICH on mo****od swings**

**Figure S17. Leave-one-out graph for ICH on ma****jor depressive disorder**

All

rs7193065

rs13140136

rs78601792

rs7589494

rs2571227

rs12318133

rs72839026

−0.2

0.0

0.2

0.4

**Figure S18. Leave-one-out graph for ICH on atte****ntion deficit/hyperactivity disorder**

All

rs72839026

rs2571227

rs7589494

rs9835089

rs12318133

rs13140136

rs75534956

−20

−10

0

10

20

30

MR leave−one−out sensitivity analysis for


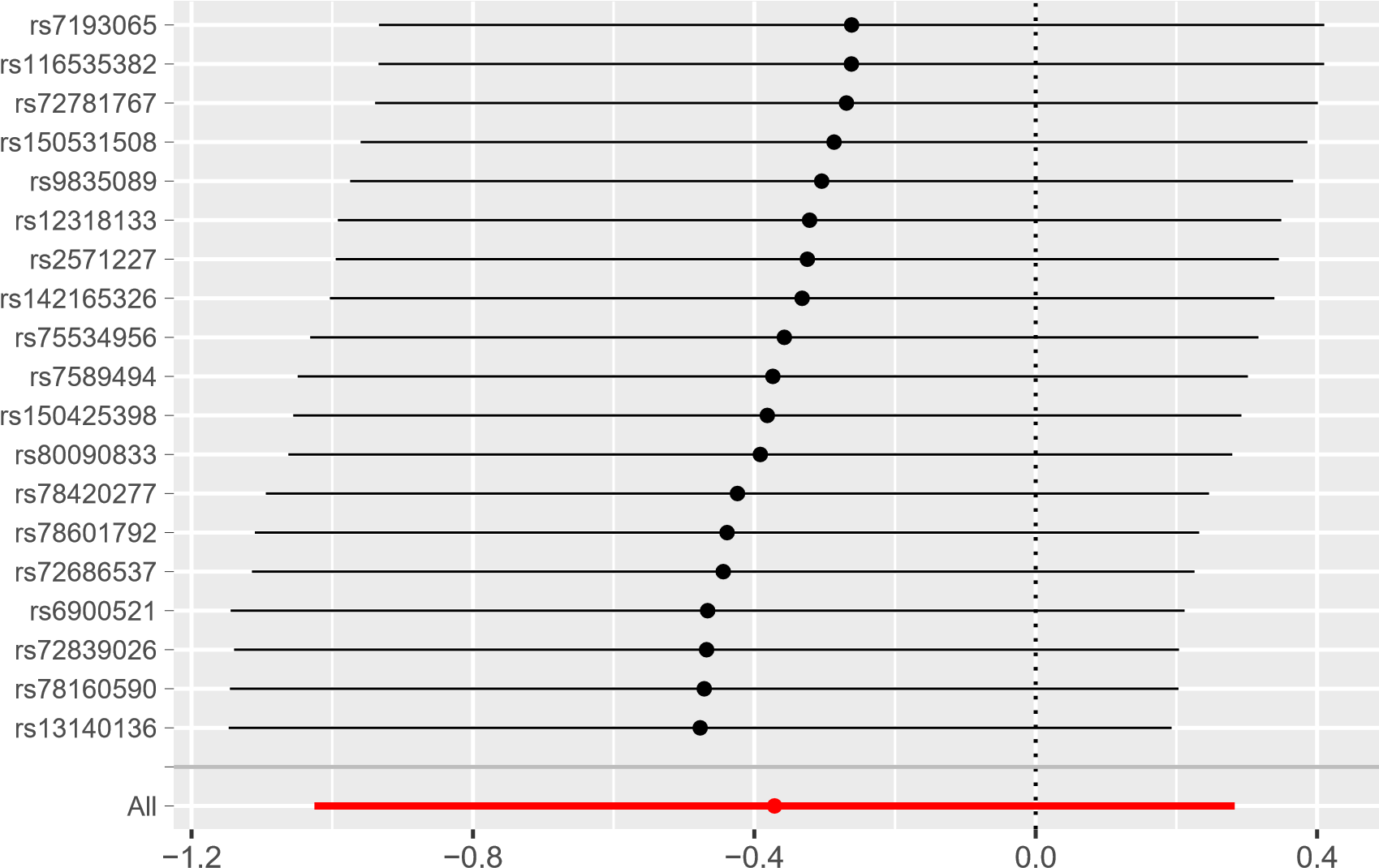
**Figure S19. Leave-one-out graph for ICH on an****xiety**

**Figure S20. Leave-one-out graph for ICH on ins****omnia**

**
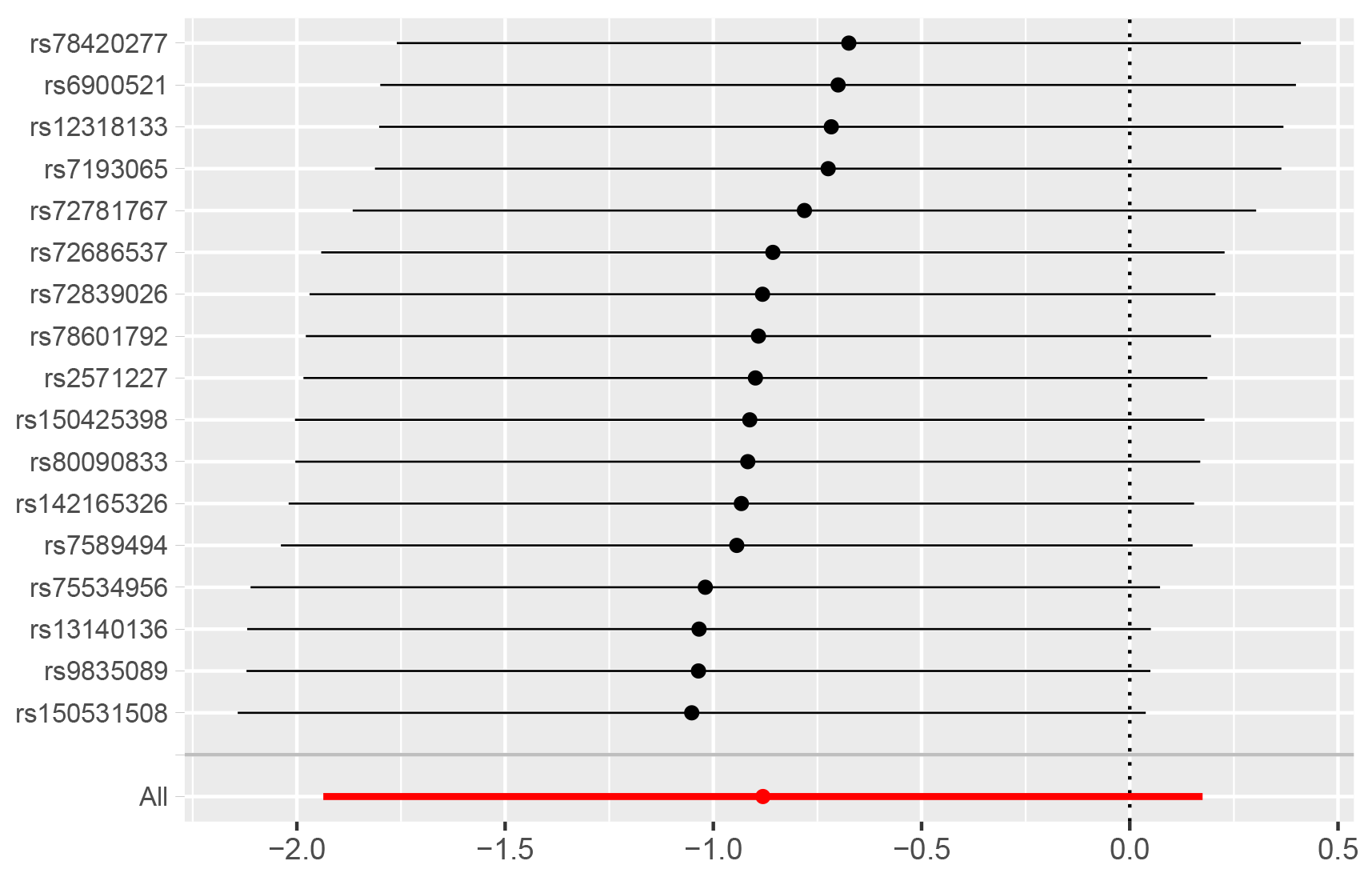
**


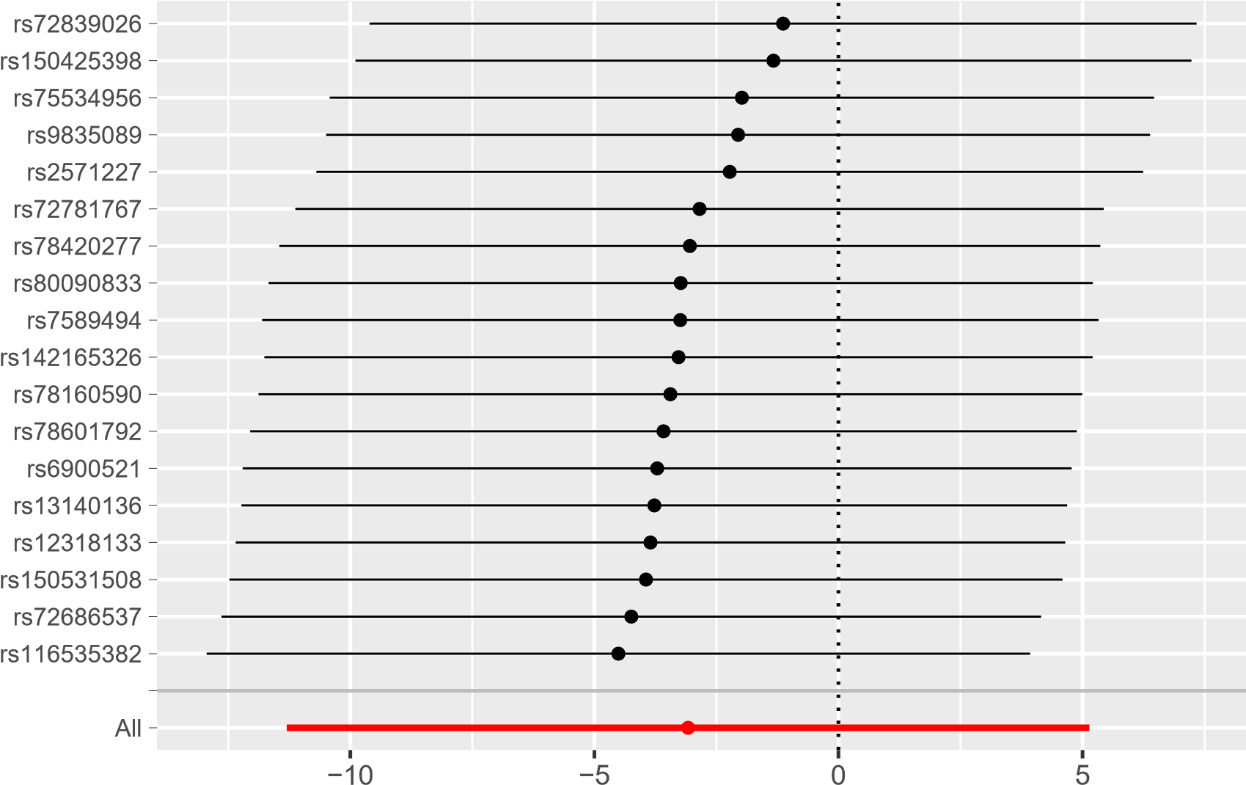
**Figure S21. Leave-one-out graph for ICH on sch****izophrenia**


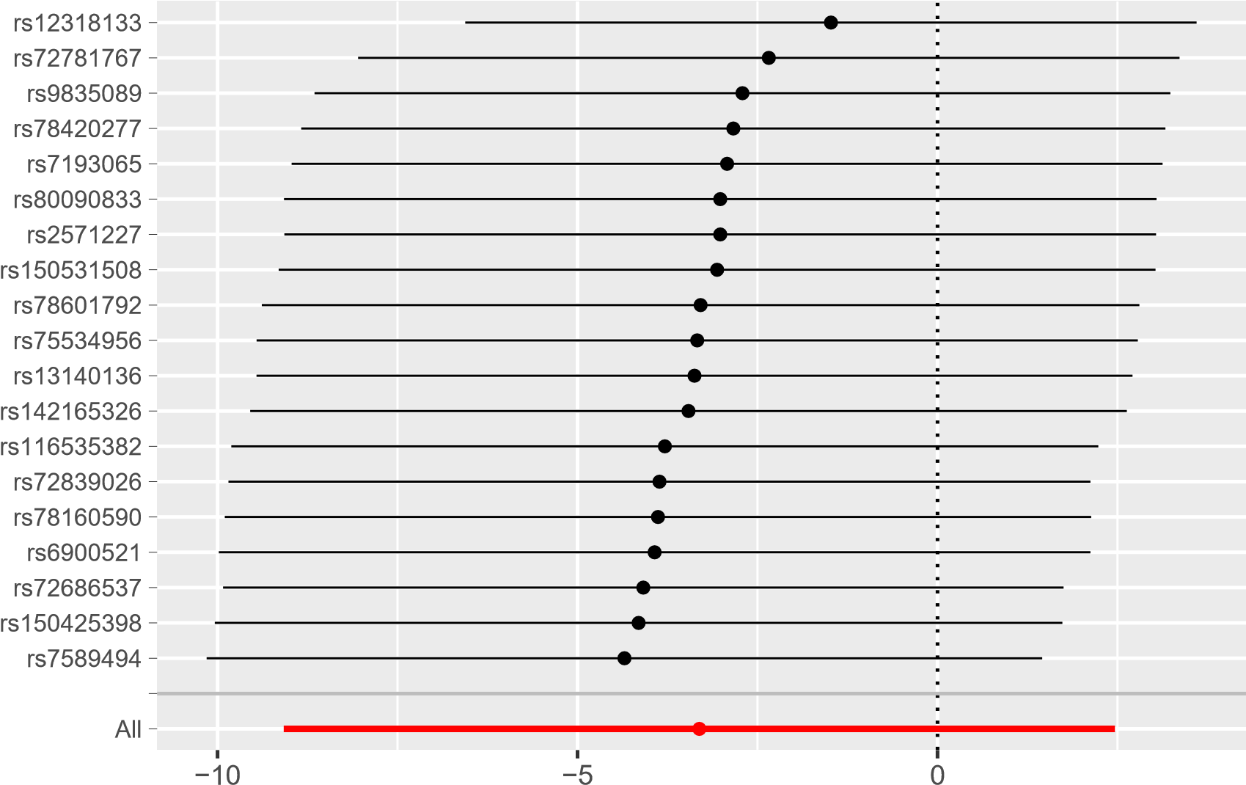
**Figure S22. Leave-one-out graph for ICH on neu****roticism**


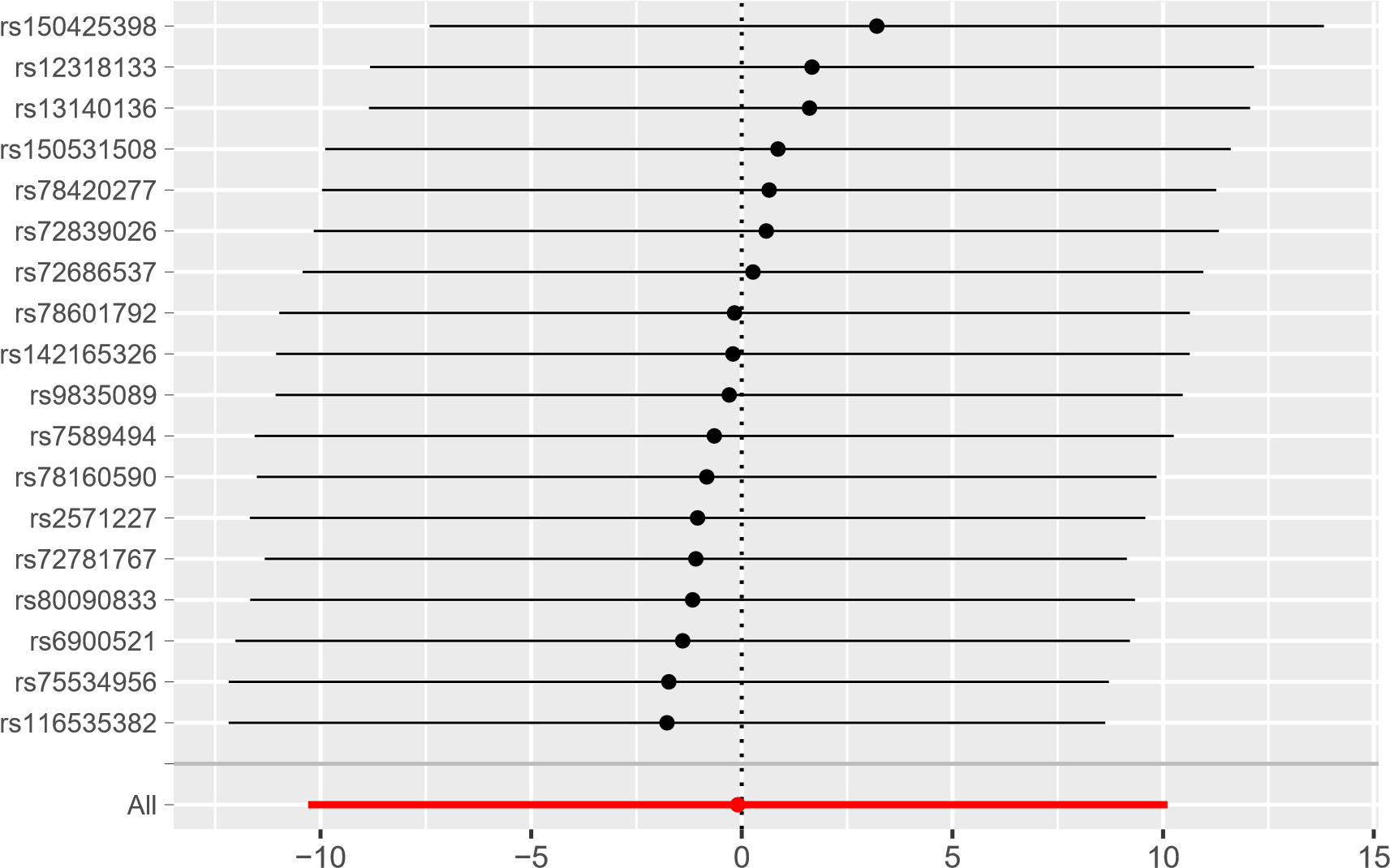
**Figure S23. Leave-one-out graph for ICH on bip****olar disorder**
