# Supplementary material for: Psychiatric traits and intracerebral hemorrhage: A Mendelian randomization study
Source: Front Psychiatry. 2023 Jan 4;13:1049432. doi: 10.3389/fpsyt.2022.1049432 (PMC9850495; doi:10.3389/fpsyt.2022.1049432)
Supplement: Supplementary file 2 [file Table_1.docx]

| **Index of supplementary Table S1-S16** |  |
| --- | --- |
| [**Table S1.** The definition and MR base ID of the eight psychiatric traits and ICH](#S1) | Page 1 |
| [**Table S2.** 56 valid IVs used for MR analysis of mood swings on ICH](#S2) | Page 2 |
| [**Table S3.** 32 valid IVs used for MR analysis of major depressive disorder on ICH](#S3) | Page 4 |
| [**Table S4.** 9 valid IVs used for MR analysis of attention deficit/hyperactivity disorder on ICH](#S4) | Page 5 |
| [**Table S5.** 40 valid IVs used for MR analysis of anxiety on ICH](#S5) | Page 6 |
| [**Table S6.** 36 valid IVs used for MR analysis of insomnia on ICH](#S6) | Page 7 |
| [**Table S7.** 71 valid IVs used for MR analysis of schizophrenia on ICH](#S7) | Page 8 |
| [**Table S8.** 109 valid IVs used for MR analysis of neuroticism on ICH](#S8) | Page 10 |
| [**Table S9.** 13 valid IVs used for MR analysis of bipolar disorder on ICH](#S9) | Page 13 |
| [**Table S10.** Details of SNPs that may be associated with confounding factor phenotypes](#S18) | Page 14 |
| [**Table S11.** 19 valid IVs used for MR analysis of ICH on mood swings](#S10) | Page 15 |
| [**Table S12. 7** valid IVs used for MR analysis of ICH on major depressive disorder](#S11) | Page 16 |
| [**Table S13.** 7 valid IVs used for MR analysis of ICH on attention deficit/hyperactivity disorder](#S12) | Page 17 |
| [**Table S14.** 19 valid IVs used for MR analysis of ICH on anxiety](#S13) | Page 18 |
| [**Table S15.** 17 valid IVs used for MR analysis of ICH on insomnia](#S14) | Page 19 |
| [**Table S16.** 18 valid IVs used for MR analysis of ICH on schizophrenia](#S15) | Page 20 |
| [**Table S17.** 19 valid IVs used for MR analysis of ICH on neuroticism](#S16) | Page 21 |
| [**Table S18.** 18 valid IVs used for MR analysis of ICH on bipolar disorder](#S17) | Page 22 |

**Table S1. The definition and MR base ID** **of the eight psychiatric traits and ICH**

| Variables (MR base ID) | Definition |
| --- | --- |
| Mood swings (ukb-b-14180) | Rapid fluctuations in strong emotions and difficulty in regulating these swings or their behavioral consequences |
| Major depressive disorder (ieu-a-1187) | Major depressive disorder is a mood dysfunction caused by a genetic abnormality or a dramatic change in the patient's environment, with a series of depressive symptoms dominated by persistent spontaneous depression |
| Attention-deficit/hyperactivity disorder (ieu-a-1183) | A group of syndromes that occur in childhood and are characterized by significant attentional difficulties, short attention span, hyperactivity or impulsivity compared to children of the same age |
| Anxiety (ukb-b-6991) | A future-oriented emotional state related to the preparation for possible, imminent negative events |
| Insomnia (ukb-b-3957) | Insomnia is mainly characterized by dissatisfaction with the duration or quality of sleep, the presence of difficulty falling asleep or maintaining sleep, and the impact on daytime functioning |
| Schizophrenia (ieu-b-42) | Presence of persistent or episodic delusions, hallucinations, disorganized thinking, grossly disorganized behavior, and experiences of passivity and control, negative symptoms such as blunted or flat affect and avolition, and psychomotor disturbances |
| Neuroticism (ukb-b-4630) | Tendency to experience a broad range of distressing emotions such as anxiety, anger irritability, depression, and other negative emotional states, often in response to even relatively minor actual or perceived stressors |
| Bipolar disorder (ieu-b-41) | Circular Insanity, with manic and depressive periods as well as intervening periods of euthymia or illness-free intervals |
| Intracerebral hemorrhage (ukb-d-I9_INTRACRA) | The phenotype used for ICH is defined according to the ICD-10 codes. It corresponds to the classification of I61 Intracerebral hemorrhage |

**Table S2. 56 valid IVs use****d for MR analysis of mood swings on ICH**

| SNP | Effect allele | Non-effect allele | Effect allele frequency | Beta | SE | *p* value | F Statistics |
| --- | --- | --- | --- | --- | --- | --- | --- |
| rs10210512 | G | T | 0.4201 | 0.0064 | 0.0011 | 1.30E-09 | 36.80 |
| rs1050863 | A | G | 0.5666 | -0.0076 | 0.0011 | 5.80E-13 | 51.90 |
| rs1055710 | A | G | 0.3317 | -0.0066 | 0.0011 | 2.30E-09 | 35.67 |
| rs10983775 | T | C | 0.5344 | -0.0069 | 0.0010 | 5.10E-11 | 43.14 |
| rs11168048 | C | T | 0.4190 | -0.0061 | 0.0011 | 6.30E-09 | 33.75 |
| rs11184994 | T | C | 0.6941 | 0.0064 | 0.0011 | 1.20E-08 | 32.53 |
| rs11599236 | C | T | 0.4111 | -0.0063 | 0.0011 | 3.60E-09 | 34.83 |
| rs11665070 | A | G | 0.6671 | -0.0084 | 0.0011 | 2.60E-14 | 58.00 |
| rs11720128 | T | C | 0.4477 | -0.0060 | 0.0011 | 3.50E-08 | 30.39 |
| rs11728841 | C | A | 0.5558 | 0.0058 | 0.0011 | 2.90E-08 | 30.80 |
| rs12530421 | C | A | 0.0667 | 0.0122 | 0.0021 | 5.20E-09 | 32.76 |
| rs12963231 | A | C | 0.3294 | 0.0081 | 0.0011 | 3.50E-13 | 52.90 |
| rs13085679 | A | G | 0.5007 | 0.0069 | 0.0010 | 4.20E-11 | 43.52 |
| rs13434208 | G | A | 0.5251 | -0.0060 | 0.0010 | 7.60E-09 | 33.39 |
| rs1439252 | A | G | 0.3732 | -0.0066 | 0.0011 | 1.30E-09 | 36.82 |
| rs1688000 | G | A | 0.6254 | -0.0063 | 0.0011 | 4.40E-09 | 34.44 |
| rs1724411 | C | T | 0.2293 | 0.0117 | 0.0012 | 4.20E-21 | 88.86 |
| rs17411061 | T | C | 0.4195 | 0.0070 | 0.0011 | 4.10E-11 | 43.56 |
| rs1962104 | C | T | 0.5581 | 0.0073 | 0.0011 | 5.50E-12 | 47.49 |
| rs2000228 | C | T | 0.3133 | 0.0071 | 0.0011 | 2.10E-10 | 40.38 |
| rs2016851 | C | T | 0.7035 | 0.0063 | 0.0012 | 4.50E-08 | 29.91 |
| rs2483509 | A | G | 0.4499 | 0.0064 | 0.0010 | 8.40E-10 | 37.67 |
| rs2503775 | G | A | 0.8708 | -0.0090 | 0.0016 | 6.40E-09 | 33.71 |
| rs2678897 | A | G | 0.6075 | 0.0068 | 0.0011 | 2.50E-10 | 40.04 |
| rs28517342 | G | T | 0.2567 | -0.0066 | 0.0012 | 3.50E-08 | 30.41 |
| rs28655666 | A | G | 0.5523 | -0.0070 | 0.0010 | 2.40E-11 | 44.65 |
| rs297343 | G | T | 0.6378 | -0.0073 | 0.0011 | 2.10E-11 | 44.83 |
| rs3012850 | G | C | 0.2393 | 0.0093 | 0.0012 | 6.80E-14 | 56.13 |
| rs35789697 | A | G | 0.3694 | -0.0067 | 0.0011 | 5.00E-10 | 38.68 |
| rs35856211 | A | G | 0.5398 | -0.0066 | 0.0011 | 4.40E-10 | 38.92 |
| rs4243048 | A | G | 0.5574 | -0.0064 | 0.0011 | 2.30E-09 | 35.73 |
| rs4309187 | C | A | 0.6821 | 0.0090 | 0.0011 | 1.40E-15 | 63.74 |
| rs4836789 | C | T | 0.5963 | 0.0072 | 0.0011 | 1.50E-11 | 45.56 |
| rs4899532 | G | A | 0.7452 | 0.0078 | 0.0012 | 6.40E-11 | 42.69 |
| rs55774086 | G | C | 0.2429 | 0.0077 | 0.0012 | 2.50E-10 | 40.03 |
| rs56116032 | G | A | 0.2194 | -0.0092 | 0.0013 | 2.40E-13 | 53.63 |
| rs600011 | C | A | 0.3014 | 0.0070 | 0.0011 | 8.40E-10 | 37.66 |
| rs6103271 | G | A | 0.8248 | -0.0091 | 0.0014 | 5.70E-11 | 42.93 |
| rs613872 | T | G | 0.8258 | 0.0109 | 0.0014 | 1.80E-15 | 63.31 |
| rs61937595 | T | C | 0.0941 | 0.0101 | 0.0018 | 2.80E-08 | 30.87 |
| rs6460902 | A | G | 0.4190 | 0.0065 | 0.0011 | 8.10E-10 | 37.74 |
| rs67447472 | T | G | 0.0996 | 0.0110 | 0.0018 | 2.80E-10 | 39.80 |
| rs67970900 | T | G | 0.3088 | -0.0067 | 0.0011 | 3.20E-09 | 35.06 |
| rs6895295 | T | C | 0.2157 | 0.0071 | 0.0013 | 2.60E-08 | 30.99 |
| rs7047280 | T | C | 0.6050 | -0.0062 | 0.0011 | 7.80E-09 | 33.33 |
| rs7202252 | C | T | 0.7324 | 0.0073 | 0.0012 | 4.90E-10 | 38.71 |
| rs72660658 | T | C | 0.2672 | -0.0068 | 0.0012 | 6.90E-09 | 33.56 |
| rs7536987 | G | A | 0.2775 | -0.0065 | 0.0012 | 1.90E-08 | 31.55 |
| rs77087420 | G | A | 0.0546 | -0.0127 | 0.0023 | 3.00E-08 | 31.67 |
| rs771998 | C | T | 0.2763 | 0.0064 | 0.0012 | 4.80E-08 | 29.78 |
| rs7818437 | C | T | 0.2362 | 0.0091 | 0.0012 | 1.80E-13 | 54.21 |
| rs7954112 | A | G | 0.4188 | 0.0059 | 0.0011 | 3.10E-08 | 30.65 |
| rs926914 | T | C | 0.2884 | 0.0081 | 0.0012 | 2.30E-12 | 49.17 |
| rs931235 | C | A | 0.5929 | -0.0061 | 0.0011 | 9.40E-09 | 32.97 |
| rs9517313 | C | G | 0.3812 | 0.0064 | 0.0011 | 1.80E-09 | 36.18 |
| rs999483 | G | T | 0.2493 | 0.0070 | 0.0012 | 7.40E-09 | 33.43 |

**Table S3. 32 valid IV****s used for MR analysis of major depressive disorder on ICH**

| SNP | Effect allele | Non-effect allele | Effect allele frequency | Beta | SE | *p* value | F Statistics |
| --- | --- | --- | --- | --- | --- | --- | --- |
| rs10149470 | G | A | 0.5147 | 0.0290 | 0.0049 | 3.05E-09 | 35.02 |
| rs10950398 | A | G | 0.4181 | 0.0275 | 0.0049 | 2.55E-08 | 31.49 |
| rs10959913 | G | T | 0.2454 | -0.0334 | 0.0057 | 5.06E-09 | 34.33 |
| rs11135349 | C | A | 0.5353 | 0.0294 | 0.0048 | 1.09E-09 | 37.51 |
| rs11643192 | A | C | 0.3866 | 0.0270 | 0.0049 | 3.36E-08 | 30.37 |
| rs11663393 | A | G | 0.4614 | 0.0278 | 0.0049 | 1.65E-08 | 32.19 |
| rs11682175 | C | T | 0.4729 | 0.0281 | 0.0048 | 4.68E-09 | 34.27 |
| rs1226412 | T | C | 0.7968 | 0.0332 | 0.0059 | 2.38E-08 | 31.67 |
| rs12552 | G | A | 0.5637 | -0.0429 | 0.0048 | 6.07E-19 | 79.87 |
| rs12666117 | A | G | 0.4639 | 0.0274 | 0.0048 | 1.35E-08 | 32.59 |
| rs12958048 | G | A | 0.6658 | -0.0338 | 0.0051 | 3.61E-11 | 43.93 |
| rs1354115 | A | C | 0.6312 | 0.0276 | 0.0049 | 2.37E-08 | 31.72 |
| rs1432639 | A | C | 0.5995 | 0.0390 | 0.0050 | 4.55E-15 | 60.84 |
| rs17727765 | C | T | 0.0812 | 0.0508 | 0.0088 | 8.51E-09 | 33.32 |
| rs1806153 | T | G | 0.2311 | 0.0361 | 0.0059 | 1.18E-09 | 37.44 |
| rs2005864 | T | C | 0.4366 | 0.0282 | 0.0049 | 6.73E-09 | 33.12 |
| rs2389016 | T | C | 0.2964 | 0.0305 | 0.0053 | 1.02E-08 | 33.12 |
| rs247910 | G | A | 0.4573 | 0.0315 | 0.0049 | 1.07E-10 | 41.33 |
| rs4074723 | C | A | 0.5891 | 0.0270 | 0.0049 | 3.12E-08 | 30.37 |
| rs4904738 | C | T | 0.4280 | 0.0289 | 0.0049 | 2.57E-09 | 34.79 |
| rs5758265 | A | G | 0.2867 | 0.0310 | 0.0054 | 7.55E-09 | 32.97 |
| rs61867293 | T | C | 0.1921 | -0.0374 | 0.0061 | 6.97E-10 | 37.59 |
| rs6905391 | A | G | 0.1642 | -0.0443 | 0.0069 | 1.35E-10 | 41.21 |
| rs7198928 | C | T | 0.3847 | -0.0284 | 0.0050 | 1.00E-08 | 32.27 |
| rs7430565 | A | G | 0.5847 | -0.0288 | 0.0048 | 2.87E-09 | 36.00 |
| rs76485002 | G | A | 0.0299 | -0.1087 | 0.0180 | 1.60E-09 | 36.47 |
| rs7856424 | T | C | 0.2838 | -0.0306 | 0.0053 | 8.48E-09 | 33.34 |
| rs8025231 | C | A | 0.4481 | 0.0339 | 0.0048 | 2.36E-12 | 49.87 |
| rs8063603 | A | G | 0.6803 | -0.0308 | 0.0053 | 6.86E-09 | 33.77 |
| rs915057 | G | A | 0.5662 | 0.0300 | 0.0049 | 7.61E-10 | 37.47 |
| rs9402472 | A | G | 0.2426 | 0.0327 | 0.0059 | 2.78E-08 | 30.72 |
| rs9427672 | G | A | 0.7603 | 0.0321 | 0.0058 | 3.12E-08 | 30.63 |

**Table S4. 9 valid IVs** **used for MR analysis of attention deficit/hyperactivity disorder on ICH**

| SNP | Effect allele | | Non-effect allele | Effect allele frequency | Beta | | SE | *p* value | F Statistics |
| --- | --- | --- | --- | --- | --- | --- | --- | --- | --- |
| rs10262192 | A | G | | 0.4760 | 0.0732 | 0.0132 | | 2.89E-08 | 30.75 |
| rs112984125 | A | G | | 0.2982 | -0.1060 | 0.0146 | | 3.58E-13 | 52.71 |
| rs1222063 | A | G | | 0.3512 | 0.0962 | 0.0174 | | 3.07E-08 | 30.57 |
| rs1427829 | G | A | | 0.5469 | -0.0799 | 0.0133 | | 1.82E-09 | 36.09 |
| rs212178 | A | G | | 0.8871 | -0.1154 | 0.0200 | | 7.68E-09 | 33.29 |
| rs28411770 | C | T | | 0.3449 | -0.0861 | 0.0151 | | 1.15E-08 | 32.51 |
| rs4858241 | G | T | | 0.3762 | -0.0789 | 0.0140 | | 1.74E-08 | 31.76 |
| rs74760947 | G | A | | 0.0488 | 0.1798 | 0.0317 | | 1.35E-08 | 32.17 |
| rs9677504 | A | G | | 0.0937 | 0.1169 | 0.0206 | | 1.39E-08 | 32.20 |

**Table S5. 40 valid IVs** **used for MR analysis of anxiety on ICH**

| SNP | Effect allele | Non-effect allele | | Effect allele frequency | Beta | SE | *p* value | F Statistics |
| --- | --- | --- | --- | --- | --- | --- | --- | --- |
| rs10035449 | C | T | 0.5333 | | 0.0059 | 0.0010 | 2.10E-09 | 35.87 |
| rs10141157 | C | T | 0.5138 | | 0.0058 | 0.0010 | 3.00E-09 | 35.19 |
| rs1021363 | G | A | 0.6436 | | -0.0074 | 0.0010 | 3.90E-13 | 52.68 |
| rs10264984 | T | C | 0.4094 | | 0.0058 | 0.0010 | 4.50E-09 | 34.40 |
| rs10762080 | G | A | 0.5849 | | -0.0056 | 0.0010 | 1.40E-08 | 32.16 |
| rs10818865 | G | A | 0.2090 | | 0.0068 | 0.0012 | 1.30E-08 | 32.27 |
| rs11123030 | C | T | 0.5109 | | -0.0060 | 0.0010 | 1.10E-09 | 37.20 |
| rs113878233 | T | C | 0.0610 | | 0.0115 | 0.0020 | 2.20E-08 | 34.25 |
| rs12159707 | A | G | 0.3442 | | -0.0059 | 0.0010 | 1.50E-08 | 32.01 |
| rs12201442 | G | A | 0.1065 | | 0.0099 | 0.0016 | 4.80E-10 | 38.77 |
| rs12919291 | C | G | 0.1892 | | 0.0073 | 0.0012 | 5.70E-09 | 33.92 |
| rs12967143 | C | G | 0.6994 | | -0.0076 | 0.0011 | 1.20E-12 | 50.53 |
| rs12967855 | G | A | 0.6688 | | -0.0069 | 0.0010 | 4.20E-11 | 43.54 |
| rs13084037 | A | G | 0.7744 | | -0.0065 | 0.0012 | 2.50E-08 | 31.08 |
| rs1536873 | A | G | 0.4690 | | 0.0056 | 0.0010 | 1.60E-08 | 31.91 |
| rs1814912 | C | T | 0.3256 | | 0.0062 | 0.0011 | 4.90E-09 | 34.23 |
| rs2283066 | C | T | 0.3755 | | -0.0057 | 0.0010 | 1.30E-08 | 32.36 |
| rs2298969 | G | A | 0.4815 | | -0.0054 | 0.0010 | 4.20E-08 | 30.04 |
| rs2698323 | C | T | 0.4157 | | 0.0055 | 0.0010 | 2.10E-08 | 31.41 |
| rs30266 | A | G | 0.3282 | | 0.0077 | 0.0010 | 1.80E-13 | 54.19 |
| rs34555420 | T | G | 0.0978 | | -0.0090 | 0.0016 | 4.20E-08 | 30.06 |
| rs3746522 | T | C | 0.2159 | | 0.0073 | 0.0012 | 1.60E-09 | 36.44 |
| rs3807866 | A | G | 0.4107 | | 0.0075 | 0.0010 | 3.90E-14 | 57.19 |
| rs4245147 | T | C | 0.5145 | | 0.0058 | 0.0010 | 3.90E-09 | 34.65 |
| rs4404022 | T | A | 0.4165 | | 0.0056 | 0.0010 | 1.80E-08 | 31.75 |
| rs4518438 | C | T | 0.5101 | | -0.0057 | 0.0010 | 6.60E-09 | 33.66 |
| rs486584 | T | C | 0.5238 | | 0.0055 | 0.0010 | 1.60E-08 | 31.94 |
| rs56116032 | G | A | 0.2195 | | -0.0066 | 0.0012 | 2.00E-08 | 31.48 |
| rs621313 | G | A | 0.4912 | | 0.0075 | 0.0010 | 2.20E-14 | 58.35 |
| rs6466512 | A | G | 0.4173 | | -0.0058 | 0.0010 | 5.60E-09 | 33.97 |
| rs66511648 | C | T | 0.2847 | | 0.0062 | 0.0011 | 1.30E-08 | 32.29 |
| rs6699744 | T | A | 0.6160 | | 0.0082 | 0.0010 | 4.30E-16 | 66.10 |
| rs67981811 | G | C | 0.1142 | | -0.0119 | 0.0015 | 1.00E-14 | 59.85 |
| rs6818069 | G | T | 0.2555 | | 0.0064 | 0.0011 | 1.20E-08 | 32.55 |
| rs7046881 | G | T | 0.4232 | | -0.0054 | 0.0010 | 4.00E-08 | 30.13 |
| rs7528182 | T | C | 0.4332 | | 0.0059 | 0.0010 | 2.70E-09 | 35.40 |
| rs7548487 | G | A | 0.1162 | | 0.0088 | 0.0015 | 7.20E-09 | 33.47 |
| rs7583068 | A | T | 0.3196 | | 0.0063 | 0.0010 | 2.40E-09 | 35.64 |
| rs9347903 | T | C | 0.2263 | | 0.0068 | 0.0012 | 5.40E-09 | 34.04 |
| rs9530139 | T | C | 0.1944 | | -0.0076 | 0.0012 | 7.90E-10 |  |

**Table S6. 36 valid IV****s used for MR analysis of insomnia on ICH**

| SNP | Effect allele | Non-effect allele | Effect allele frequency | | Beta | SE | *p* value | F Statistics |
| --- | --- | --- | --- | --- | --- | --- | --- | --- |
| rs10838708 | A | G | 0.4590 | -0.0095 | | 0.0015 | 2.90E-10 | 39.77 |
| rs11097861 | G | A | 0.7163 | 0.0100 | | 0.0016 | 1.10E-09 | 37.08 |
| rs11152363 | A | G | 0.1863 | 0.0156 | | 0.0019 | 4.50E-16 | 65.99 |
| rs113851554 | T | G | 0.0573 | 0.0468 | | 0.0033 | 2.90E-45 | 199.34 |
| rs11635495 | C | T | 0.5122 | 0.0094 | | 0.0015 | 2.80E-10 | 39.84 |
| rs11790060 | C | T | 0.3308 | -0.0103 | | 0.0016 | 5.80E-11 | 42.90 |
| rs12049261 | C | G | 0.2925 | 0.0112 | | 0.0016 | 6.80E-12 | 47.07 |
| rs12470989 | G | A | 0.2039 | -0.0102 | | 0.0018 | 2.80E-08 | 30.83 |
| rs1547630 | A | G | 0.6515 | 0.0091 | | 0.0016 | 5.80E-09 | 33.89 |
| rs1592757 | C | G | 0.3558 | 0.0102 | | 0.0015 | 4.30E-11 | 43.49 |
| rs17151854 | T | G | 0.1524 | 0.0130 | | 0.0021 | 3.80E-10 | 39.21 |
| rs17709610 | G | A | 0.2980 | -0.0099 | | 0.0016 | 9.50E-10 | 37.43 |
| rs1988337 | G | A | 0.5524 | 0.0084 | | 0.0015 | 2.10E-08 | 31.41 |
| rs2062113 | C | T | 0.5683 | -0.0096 | | 0.0015 | 1.60E-10 | 40.95 |
| rs224032 | A | G | 0.5504 | 0.0084 | | 0.0015 | 1.80E-08 | 31.67 |
| rs2604551 | G | T | 0.6404 | -0.0085 | | 0.0016 | 4.70E-08 | 29.85 |
| rs314280 | G | A | 0.5470 | 0.0097 | | 0.0015 | 7.30E-11 | 42.42 |
| rs324017 | C | A | 0.7054 | -0.0099 | | 0.0016 | 1.40E-09 | 36.69 |
| rs4572538 | T | C | 0.3641 | -0.0096 | | 0.0016 | 7.70E-10 | 37.84 |
| rs4577309 | G | A | 0.5337 | -0.0085 | | 0.0015 | 1.00E-08 | 32.82 |
| rs4886860 | C | G | 0.7674 | -0.0118 | | 0.0018 | 1.80E-11 | 45.14 |
| rs56093896 | A | C | 0.2141 | -0.0124 | | 0.0018 | 7.70E-12 | 46.84 |
| rs56330606 | G | A | 0.3790 | 0.0093 | | 0.0015 | 1.20E-09 | 37.00 |
| rs56365214 | A | C | 0.1558 | -0.0148 | | 0.0021 | 5.60E-13 | 51.97 |
| rs6561715 | A | T | 0.6307 | -0.0116 | | 0.0015 | 4.80E-14 | 56.79 |
| rs6690017 | G | T | 0.4089 | -0.0103 | | 0.0015 | 1.10E-11 | 46.22 |
| rs68094047 | T | C | 0.2513 | 0.0103 | | 0.0017 | 1.70E-09 | 36.25 |
| rs6975972 | G | A | 0.5787 | -0.0090 | | 0.0015 | 2.00E-09 | 35.96 |
| rs705219 | A | T | 0.8874 | 0.0134 | | 0.0024 | 1.20E-08 | 32.54 |
| rs72924721 | T | C | 0.0731 | 0.0165 | | 0.0029 | 1.10E-08 | 32.71 |
| rs7711696 | T | G | 0.3050 | 0.0112 | | 0.0016 | 4.10E-12 | 48.07 |
| rs931221 | A | T | 0.2367 | 0.0106 | | 0.0018 | 1.30E-09 | 36.81 |
| rs9570080 | C | T | 0.3441 | -0.0106 | | 0.0016 | 1.60E-11 | 45.41 |
| rs9845387 | A | C | 0.0403 | -0.0219 | | 0.0038 | 7.10E-09 | 33.49 |
| rs9894577 | A | G | 0.3182 | 0.0132 | | 0.0016 | 1.30E-16 | 68.38 |
| rs9906181 | G | A | 0.6876 | -0.0092 | | 0.0016 | 2.40E-08 | 31.18 |

**Table S7. 71 v****alid IVs used for MR analysis of schizophrenia on ICH**

| SNP | Effect allele | Non-effect allele | Effect allele frequency | Beta | SE | *p* value | F Statistics |
| --- | --- | --- | --- | --- | --- | --- | --- |
| rs10108725 | T | C | 0.2220 | 0.0732 | 0.0133 | 3.32E-08 | 30.29 |
| rs1024582 | G | A | 0.6600 | -0.0989 | 0.0115 | 6.27E-18 | 73.96 |
| rs10791097 | G | T | 0.5200 | -0.0766 | 0.0109 | 2.05E-12 | 49.38 |
| rs11027857 | A | G | 0.5870 | 0.0640 | 0.0109 | 3.67E-09 | 34.47 |
| rs1106568 | A | G | 0.7440 | -0.0694 | 0.0125 | 2.85E-08 | 30.83 |
| rs111294930 | G | A | 0.2190 | -0.0877 | 0.0143 | 9.29E-10 | 37.61 |
| rs11139497 | A | T | 0.3360 | 0.0656 | 0.0118 | 2.65E-08 | 30.91 |
| rs11191419 | A | T | 0.3340 | -0.1016 | 0.0118 | 6.69E-18 | 74.13 |
| rs11682175 | C | T | 0.4390 | 0.0735 | 0.0109 | 1.58E-11 | 45.46 |
| rs11693094 | T | C | 0.4290 | -0.0736 | 0.0110 | 2.17E-11 | 44.77 |
| rs11693528 | G | C | 0.1850 | 0.1028 | 0.0136 | 4.73E-14 | 57.13 |
| rs117074560 | T | C | 0.0370 | -0.1566 | 0.0277 | 1.66E-08 | 31.96 |
| rs11740474 | T | A | 0.4120 | 0.0627 | 0.0112 | 2.00E-08 | 31.33 |
| rs1191551 | G | T | 0.7840 | -0.0717 | 0.0131 | 4.21E-08 | 29.95 |
| rs12062861 | A | G | 0.1750 | -0.0911 | 0.0149 | 9.66E-10 | 37.38 |
| rs1233578 | G | A | 0.1490 | -0.1964 | 0.0164 | 6.17E-33 | 143.41 |
| rs12691307 | G | A | 0.4780 | -0.0719 | 0.0113 | 2.03E-10 | 40.49 |
| rs12704290 | A | G | 0.1310 | -0.1061 | 0.0168 | 2.59E-10 | 39.89 |
| rs12826178 | T | G | 0.0730 | -0.1682 | 0.0244 | 5.70E-12 | 47.52 |
| rs12887734 | T | G | 0.2730 | 0.0883 | 0.0121 | 3.72E-13 | 53.26 |
| rs13240464 | C | T | 0.3790 | -0.0807 | 0.0116 | 3.12E-12 | 48.40 |
| rs13261481 | G | T | 0.5910 | -0.0624 | 0.0110 | 1.66E-08 | 32.18 |
| rs1339227 | T | C | 0.3550 | -0.0633 | 0.0114 | 3.06E-08 | 30.83 |
| rs1498232 | C | T | 0.6970 | -0.0720 | 0.0118 | 1.21E-09 | 37.23 |
| rs1509378 | G | A | 0.6460 | -0.0692 | 0.0119 | 5.39E-09 | 33.81 |
| rs1615350 | T | C | 0.7340 | -0.0851 | 0.0123 | 4.26E-12 | 47.87 |
| rs16867576 | G | A | 0.1200 | -0.0958 | 0.0170 | 1.60E-08 | 31.76 |
| rs1702294 | C | T | 0.7840 | 0.1184 | 0.0138 | 1.03E-17 | 73.61 |
| rs17108967 | C | T | 0.3430 | 0.0653 | 0.0115 | 1.21E-08 | 32.24 |
| rs17194490 | T | G | 0.1650 | 0.0966 | 0.0148 | 6.38E-11 | 42.60 |
| rs2053079 | G | A | 0.2650 | 0.0718 | 0.0127 | 1.74E-08 | 31.96 |
| rs2103655 | A | G | 0.6640 | 0.0766 | 0.0119 | 1.24E-10 | 41.43 |
| rs215411 | A | T | 0.3270 | 0.0692 | 0.0115 | 1.68E-09 | 36.21 |
| rs2332700 | G | C | 0.7560 | -0.0771 | 0.0125 | 7.38E-10 | 38.04 |
| rs2414718 | A | G | 0.6170 | 0.0698 | 0.0110 | 1.98E-10 | 40.26 |
| rs2514218 | T | C | 0.3270 | -0.0722 | 0.0116 | 4.64E-10 | 38.74 |
| rs2535627 | C | T | 0.5030 | -0.0704 | 0.0109 | 1.17E-10 | 41.72 |
| rs2693698 | G | A | 0.5510 | 0.0617 | 0.0111 | 2.99E-08 | 30.90 |
| rs2905432 | A | G | 0.6710 | -0.0660 | 0.0114 | 7.51E-09 | 33.52 |
| rs2909457 | A | G | 0.5740 | -0.0597 | 0.0109 | 4.25E-08 | 29.99 |
| rs301797 | A | C | 0.3220 | 0.0661 | 0.0116 | 1.20E-08 | 32.47 |
| rs34796896 | A | G | 0.1940 | -0.0822 | 0.0135 | 1.23E-09 | 37.07 |
| rs35324223 | G | A | 0.1810 | 0.0920 | 0.0145 | 2.04E-10 | 40.25 |
| rs35998080 | T | G | 0.4500 | 0.0690 | 0.0112 | 6.95E-10 | 37.96 |
| rs36068923 | G | A | 0.1870 | 0.0835 | 0.0134 | 4.14E-10 | 38.83 |
| rs3798869 | A | G | 0.4640 | -0.0668 | 0.0110 | 1.09E-09 | 36.88 |
| rs3849046 | T | C | 0.5080 | 0.0625 | 0.0109 | 1.04E-08 | 32.87 |
| rs4129585 | C | A | 0.5580 | -0.0793 | 0.0109 | 3.61E-13 | 52.93 |
| rs4391122 | G | A | 0.4620 | 0.0780 | 0.0109 | 8.90E-13 | 51.21 |
| rs4523957 | T | G | 0.6030 | 0.0697 | 0.0115 | 1.40E-09 | 36.74 |
| rs4648845 | T | C | 0.4790 | 0.0672 | 0.0119 | 1.74E-08 | 31.89 |
| rs4766428 | T | C | 0.4590 | 0.0694 | 0.0112 | 6.12E-10 | 38.39 |
| rs55775495 | T | C | 0.7010 | -0.0690 | 0.0114 | 1.56E-09 | 36.63 |
| rs58120505 | C | T | 0.4120 | -0.0822 | 0.0111 | 1.26E-13 | 54.83 |
| rs5995756 | C | T | 0.5400 | -0.0725 | 0.0109 | 2.91E-11 | 44.24 |
| rs6439649 | T | G | 0.5620 | 0.0710 | 0.0111 | 1.37E-10 | 40.91 |
| rs6704768 | A | G | 0.5290 | -0.0766 | 0.0109 | 2.06E-12 | 49.38 |
| rs72986630 | T | C | 0.0540 | 0.1459 | 0.0266 | 4.12E-08 | 30.08 |
| rs73036062 | A | G | 0.2100 | -0.0891 | 0.0135 | 3.68E-11 | 43.56 |
| rs73191547 | T | A | 0.2860 | 0.0669 | 0.0115 | 6.13E-09 | 33.84 |
| rs75968099 | T | C | 0.3510 | 0.0801 | 0.0114 | 2.31E-12 | 49.37 |
| rs7601312 | G | A | 0.4840 | 0.0590 | 0.0108 | 4.67E-08 | 29.84 |
| rs760648 | A | G | 0.4160 | 0.0758 | 0.0118 | 1.27E-10 | 41.27 |
| rs76355118 | G | A | 0.0410 | 0.1544 | 0.0278 | 2.78E-08 | 30.85 |
| rs77149735 | A | G | 0.0160 | 0.2845 | 0.0485 | 4.40E-09 | 34.41 |
| rs7801375 | G | A | 0.8470 | 0.0830 | 0.0150 | 2.88E-08 | 30.62 |
| rs7819570 | T | G | 0.2070 | 0.0765 | 0.0140 | 4.47E-08 | 29.86 |
| rs7893279 | G | T | 0.1250 | -0.1124 | 0.0175 | 1.24E-10 | 41.25 |
| rs8055219 | A | G | 0.2590 | 0.0770 | 0.0127 | 1.45E-09 | 36.76 |
| rs832190 | T | C | 0.6130 | -0.0699 | 0.0113 | 5.73E-10 | 38.26 |
| rs9636107 | G | A | 0.4970 | 0.0796 | 0.0108 | 2.17E-13 | 54.31 |

**Table S8. 109 valid IVs used for MR analysis of neuroticism on ICH**

| SNP | Effect allele | Non-effect allele | Effect allele frequency | Beta | SE | *p* value | F Statistics |
| --- | --- | --- | --- | --- | --- | --- | --- |
| rs1002655 | G | C | 0.7062 | -0.0570 | 8.13E-03 | 2.30E-12 | 49.17 |
| rs10032297 | T | A | 0.6019 | 0.0462 | 7.58E-03 | 1.10E-09 | 37.10 |
| rs10045508 | G | T | 0.2198 | 0.0518 | 8.95E-03 | 7.20E-09 | 33.47 |
| rs10119773 | G | A | 0.5369 | 0.0493 | 7.49E-03 | 4.80E-11 | 43.26 |
| rs10172342 | G | A | 0.2524 | 0.0471 | 8.51E-03 | 3.20E-08 | 30.56 |
| rs1019706 | G | A | 0.2993 | -0.0525 | 8.09E-03 | 8.20E-11 | 42.20 |
| rs10456089 | A | G | 0.0787 | -0.0843 | 1.43E-02 | 3.80E-09 | 34.75 |
| rs10476484 | G | A | 0.2673 | 0.0494 | 8.35E-03 | 3.20E-09 | 35.05 |
| rs10745624 | C | T | 0.4540 | 0.0436 | 7.47E-03 | 5.30E-09 | 34.09 |
| rs10812851 | C | T | 0.3661 | -0.0478 | 7.67E-03 | 4.80E-10 | 38.77 |
| rs10852673 | A | G | 0.7291 | -0.0463 | 8.35E-03 | 3.00E-08 | 30.74 |
| rs11090045 | A | G | 0.3074 | 0.0589 | 8.11E-03 | 4.00E-13 | 52.66 |
| rs1109027 | G | A | 0.2263 | -0.0555 | 8.83E-03 | 3.10E-10 | 39.60 |
| rs113199002 | C | G | 0.3998 | -0.0417 | 7.55E-03 | 3.20E-08 | 30.57 |
| rs11509880 | A | G | 0.3301 | 0.0446 | 7.84E-03 | 1.30E-08 | 32.33 |
| rs11603808 | T | C | 0.5793 | -0.0526 | 7.48E-03 | 2.00E-12 | 49.47 |
| rs11627348 | A | C | 0.1512 | 0.0650 | 1.04E-02 | 4.20E-10 | 39.01 |
| rs11633354 | C | T | 0.5205 | -0.0458 | 7.41E-03 | 6.20E-10 | 38.26 |
| rs11665070 | A | G | 0.6680 | -0.0707 | 7.87E-03 | 2.70E-19 | 80.62 |
| rs117298864 | A | G | 0.0453 | 0.1015 | 1.81E-02 | 1.90E-08 | 31.60 |
| rs12127965 | A | G | 0.4270 | -0.0463 | 7.50E-03 | 6.50E-10 | 38.15 |
| rs1226413 | G | C | 0.7949 | 0.0617 | 9.15E-03 | 1.50E-11 | 45.50 |
| rs12466146 | C | T | 0.5758 | -0.0444 | 7.48E-03 | 3.00E-09 | 35.20 |
| rs12530421 | C | A | 0.0661 | 0.0878 | 1.48E-02 | 3.20E-09 | 35.05 |
| rs12601333 | G | C | 0.3709 | -0.0421 | 7.71E-03 | 4.80E-08 | 29.80 |
| rs12938775 | A | G | 0.5029 | -0.0492 | 7.40E-03 | 3.10E-11 | 44.10 |
| rs13226841 | C | T | 0.4873 | 0.0524 | 7.39E-03 | 1.30E-12 | 50.37 |
| rs13239186 | T | C | 0.3023 | 0.0470 | 8.10E-03 | 6.60E-09 | 33.64 |
| rs1372517 | A | G | 0.5190 | 0.0433 | 7.39E-03 | 4.70E-09 | 34.32 |
| rs1431071 | T | G | 0.2927 | -0.0503 | 8.13E-03 | 6.00E-10 | 38.31 |
| rs1542212 | G | T | 0.3923 | 0.0536 | 7.59E-03 | 1.70E-12 | 49.83 |
| rs1563245 | G | T | 0.4018 | 0.0473 | 7.58E-03 | 4.30E-10 | 38.95 |
| rs167915 | T | A | 0.3592 | -0.0477 | 7.73E-03 | 7.10E-10 | 37.99 |
| rs17096778 | G | A | 0.0476 | -0.0988 | 1.77E-02 | 2.40E-08 | 31.11 |
| rs17583539 | G | A | 0.2286 | 0.0499 | 8.91E-03 | 2.10E-08 | 31.42 |
| rs1782170 | C | A | 0.7240 | 0.0489 | 8.31E-03 | 3.90E-09 | 34.66 |
| rs1892350 | G | A | 0.4967 | 0.0441 | 7.39E-03 | 2.30E-09 | 35.68 |
| rs1903844 | C | T | 0.3567 | -0.0487 | 7.76E-03 | 3.40E-10 | 39.41 |
| rs1940735 | G | T | 0.2521 | 0.0561 | 8.53E-03 | 4.80E-11 | 43.24 |
| rs1962104 | C | T | 0.5576 | 0.0414 | 7.51E-03 | 3.50E-08 | 30.40 |
| rs2015971 | T | C | 0.4457 | -0.0424 | 7.46E-03 | 1.30E-08 | 32.33 |
| rs2042395 | A | G | 0.7710 | -0.0539 | 8.80E-03 | 9.40E-10 | 37.44 |
| rs2071754 | T | C | 0.7982 | -0.0665 | 9.20E-03 | 4.80E-13 | 52.28 |
| rs2094580 | T | G | 0.7172 | -0.0570 | 8.25E-03 | 4.90E-12 | 47.72 |
| rs2199036 | T | C | 0.3729 | -0.0467 | 7.64E-03 | 1.00E-09 | 37.30 |
| rs2278609 | C | T | 0.2180 | 0.0576 | 8.98E-03 | 1.40E-10 | 41.15 |
| rs2280711 | T | C | 0.1564 | 0.0629 | 1.02E-02 | 6.30E-10 | 38.23 |
| rs2407746 | G | C | 0.3052 | 0.0498 | 8.06E-03 | 6.60E-10 | 38.14 |
| rs240788 | G | T | 0.5725 | 0.0426 | 7.49E-03 | 1.40E-08 | 32.25 |
| rs2488401 | T | C | 0.2068 | -0.0552 | 9.19E-03 | 2.00E-09 | 36.02 |
| rs2717043 | T | C | 0.6232 | 0.0509 | 7.63E-03 | 2.60E-11 | 44.42 |
| rs2721939 | T | C | 0.6009 | 0.0421 | 7.56E-03 | 2.60E-08 | 30.98 |
| rs2791459 | A | C | 0.4106 | -0.0468 | 7.52E-03 | 4.70E-10 | 38.81 |
| rs28427480 | C | A | 0.0939 | 0.0892 | 1.27E-02 | 2.40E-12 | 49.12 |
| rs2883027 | G | A | 0.4005 | 0.0425 | 7.53E-03 | 1.70E-08 | 31.76 |
| rs2921036 | C | T | 0.5087 | -0.0826 | 7.42E-03 | 9.20E-29 | 123.83 |
| rs297343 | G | T | 0.6377 | -0.0498 | 7.71E-03 | 1.10E-10 | 41.68 |
| rs34021161 | T | C | 0.5513 | 0.0436 | 7.45E-03 | 5.00E-09 | 34.18 |
| rs34668726 | G | C | 0.1683 | 0.0595 | 9.90E-03 | 1.80E-09 | 36.19 |
| rs35267052 | G | T | 0.1028 | 0.0780 | 1.22E-02 | 1.60E-10 | 40.91 |
| rs35627347 | C | T | 0.5052 | -0.0412 | 7.55E-03 | 4.80E-08 | 29.79 |
| rs35641442 | A | G | 0.4626 | 0.0598 | 7.42E-03 | 7.60E-16 | 64.97 |
| rs36006259 | T | C | 0.1576 | -0.0579 | 1.03E-02 | 2.00E-08 | 31.53 |
| rs3772912 | G | A | 0.3621 | -0.0439 | 7.69E-03 | 1.10E-08 | 32.59 |
| rs3785232 | T | C | 0.6732 | 0.0550 | 7.95E-03 | 4.60E-12 | 47.86 |
| rs3849470 | C | T | 0.5237 | -0.0437 | 7.40E-03 | 3.40E-09 | 34.94 |
| rs4132462 | T | C | 0.3071 | 0.0449 | 8.02E-03 | 2.30E-08 | 31.25 |
| rs4140799 | A | G | 0.5321 | -0.0456 | 7.43E-03 | 8.00E-10 | 37.75 |
| rs4267163 | A | G | 0.6718 | 0.0431 | 7.88E-03 | 4.60E-08 | 29.88 |
| rs4523180 | G | T | 0.0960 | -0.0686 | 1.25E-02 | 4.50E-08 | 29.91 |
| rs4578918 | C | T | 0.7383 | -0.0508 | 8.43E-03 | 1.70E-09 | 36.28 |
| rs4585149 | C | T | 0.8200 | 0.0572 | 9.68E-03 | 3.60E-09 | 34.85 |
| rs4673866 | G | A | 0.8608 | 0.0671 | 1.07E-02 | 3.30E-10 | 39.51 |
| rs4899292 | G | A | 0.6471 | 0.0488 | 7.74E-03 | 2.90E-10 | 39.72 |
| rs55970317 | G | T | 0.3985 | -0.0523 | 7.53E-03 | 3.80E-12 | 48.23 |
| rs56116032 | G | A | 0.2198 | -0.0629 | 8.94E-03 | 1.90E-12 | 49.57 |
| rs56226325 | T | C | 0.1557 | -0.0610 | 1.02E-02 | 2.10E-09 | 35.85 |
| rs57506932 | T | C | 0.1240 | -0.0667 | 1.12E-02 | 3.10E-09 | 35.14 |
| rs59970005 | T | C | 0.2034 | -0.0605 | 9.23E-03 | 5.50E-11 | 43.01 |
| rs61038084 | G | A | 0.2775 | -0.0460 | 8.24E-03 | 2.30E-08 | 31.23 |
| rs6503091 | A | G | 0.6053 | 0.0440 | 7.58E-03 | 6.60E-09 | 33.65 |
| rs6530964 | T | C | 0.6945 | 0.0520 | 8.08E-03 | 1.20E-10 | 41.41 |
| rs6545135 | T | C | 0.3245 | -0.0431 | 7.90E-03 | 4.70E-08 | 29.83 |
| rs6606710 | C | T | 0.4064 | 0.0616 | 7.79E-03 | 2.70E-15 | 62.49 |
| rs6916891 | T | G | 0.1190 | 0.0701 | 1.15E-02 | 9.90E-10 | 37.33 |
| rs7027172 | A | G | 0.0238 | -0.1470 | 2.47E-02 | 2.50E-09 | 35.53 |
| rs703409 | T | C | 0.2519 | -0.0504 | 8.59E-03 | 4.20E-09 | 34.51 |
| rs7093157 | T | G | 0.2986 | -0.0442 | 8.09E-03 | 4.60E-08 | 29.86 |
| rs7111031 | A | C | 0.6320 | 0.0700 | 7.66E-03 | 6.80E-20 | 83.38 |
| rs7193453 | T | C | 0.4698 | -0.0415 | 7.40E-03 | 2.00E-08 | 31.46 |
| rs7235757 | A | G | 0.3133 | 0.0626 | 8.03E-03 | 6.60E-15 | 60.73 |
| rs72932350 | C | T | 0.1580 | 0.0565 | 1.01E-02 | 2.40E-08 | 31.13 |
| rs7502590 | G | A | 0.1487 | -0.0723 | 1.04E-02 | 3.40E-12 | 48.45 |
| rs753044 | T | C | 0.4136 | -0.0412 | 7.55E-03 | 4.90E-08 | 29.74 |
| rs7582403 | G | A | 0.4238 | 0.0617 | 7.49E-03 | 1.70E-16 | 67.90 |
| rs7696796 | A | G | 0.2477 | 0.0486 | 8.55E-03 | 1.30E-08 | 32.31 |
| rs77867423 | G | C | 0.0678 | -0.0852 | 1.50E-02 | 1.30E-08 | 32.35 |
| rs7869969 | G | A | 0.3317 | -0.0495 | 7.85E-03 | 2.90E-10 | 39.77 |
| rs836927 | A | C | 0.4280 | 0.0513 | 7.52E-03 | 8.60E-12 | 46.62 |
| rs872576 | T | C | 0.3342 | 0.0428 | 7.83E-03 | 4.50E-08 | 29.91 |
| rs877995 | A | G | 0.1934 | 0.0605 | 9.35E-03 | 9.70E-11 | 41.88 |
| rs910187 | A | G | 0.3736 | -0.0435 | 7.66E-03 | 1.40E-08 | 32.23 |
| rs9298995 | A | G | 0.3994 | -0.0451 | 7.55E-03 | 2.40E-09 | 35.62 |
| rs931235 | C | A | 0.5930 | -0.0464 | 7.54E-03 | 7.50E-10 | 37.87 |
| rs9341575 | C | A | 0.3088 | 0.0458 | 8.01E-03 | 1.10E-08 | 32.63 |
| rs9516861 | A | T | 0.1273 | 0.0637 | 1.11E-02 | 1.10E-08 | 32.65 |
| rs9517313 | C | G | 0.3816 | 0.0434 | 7.61E-03 | 1.10E-08 | 32.57 |
| rs9845443 | T | C | 0.7254 | 0.0474 | 8.28E-03 | 1.00E-08 | 32.77 |
| rs9908167 | A | G | 0.3951 | -0.0419 | 7.64E-03 | 4.20E-08 | 30.06 |

**Table S9. 13 v****alid IVs used for MR analysis of bipolar disorder on ICH**

| SNP | Effect allele | Non-effect allele | Effect allele frequency | Beta | SE | *p* value | F Statistics |
| --- | --- | --- | --- | --- | --- | --- | --- |
| rs10744560 | T | C | 0.3423 | 0.0832 | 0.0140 | 2.92E-09 | 35.32 |
| rs111444407 | T | C | 0.1547 | 0.1166 | 0.0184 | 2.40E-10 | 40.16 |
| rs11724116 | T | C | 0.1553 | -0.1041 | 0.0188 | 3.27E-08 | 30.66 |
| rs13231398 | C | G | 0.1071 | -0.1207 | 0.0219 | 3.36E-08 | 30.37 |
| rs17150022 | C | T | 0.1207 | 0.1132 | 0.0204 | 2.70E-08 | 30.79 |
| rs2071044 | T | C | 0.4679 | -0.0777 | 0.0135 | 9.09E-09 | 33.13 |
| rs2314398 | G | C | 0.3196 | -0.0841 | 0.0144 | 5.92E-09 | 34.11 |
| rs329319 | G | A | 0.5657 | -0.0788 | 0.0139 | 1.54E-08 | 32.14 |
| rs55648125 | G | A | 0.1089 | 0.1171 | 0.0215 | 4.92E-08 | 29.66 |
| rs71395455 | G | A | 0.3115 | -0.0821 | 0.0146 | 1.93E-08 | 31.62 |
| rs73496688 | A | T | 0.1491 | 0.1087 | 0.0190 | 1.05E-08 | 32.73 |
| rs884301 | T | C | 0.3811 | 0.0803 | 0.0138 | 5.80E-09 | 33.86 |
| rs9834970 | C | T | 0.5012 | 0.1010 | 0.0134 | 5.53E-14 | 56.81 |

**Table S10. Deta****ils of SNPs that may be associated with confounding factor phenotypes**

| **Psychiatric traits** | **SNP** | **Chr** | **Gene** | **Diseases & traits** |
| --- | --- | --- | --- | --- |
| Mood swings | rs11039154 | 11 | NR1H3 | Hypertension |
| MDD | rs159963 | 1 | RERE | Hypertension |
| ADHD | rs4916723 | 5 | LINC00461 | Alcohol consumption |
| Insomnia | rs1430205 | 5 | TMEM161B-AS1 | Alcohol consumption |
|  | rs2014830 | 3 | SEMA3F-AS1 | Diabetes |
|  | rs2297787 | 10 | CNNM2 | Hypertension |
| Schizophrenia | rs11210892 | 1 | PTPRF | Smoking |
|  | rs28681284 | 15 | CHRNA3 | Smoking |
|  | rs35225200 | 4 | MANBA | Alcohol consumption |
|  | rs4702 | 15 | FURIN | Hypertension |
|  | rs73229090 | 8 | GULOP | Alcohol consumption |
| neuroticism | rs17652520 | 17 | MAPT | Alcohol consumption |
|  | rs2269426 | 6 | TNXB | Hypertension |
|  | rs674094 | 11 | OR5AZ1P | Alcohol consumption |
|  | rs7107356 | 11 | AGBL2 | Alcohol consumption; Hypertension |
|  | rs77335224 | 10 | AS3MT | Hypertension |
| BPD | rs174592 | 11 | FADS2 | Triglycerides |

MDD, Major depressive disorder; ADHD, Attention deficit/Hyperactivity disorder; BPD, Bipolar disorder

**Table S11. 19 v****alid IVs used for MR analysis of ICH on mood swings**

| SNP | Effect allele | Non-effect allele | Effect allele frequency | Beta | SE | *p* value | F Statistics |
| --- | --- | --- | --- | --- | --- | --- | --- |
| rs116535382 | G | C | 0.0124 | 0.0032 | 0.0007 | 9.55E-07 | 24.02 |
| rs12318133 | G | A | 0.0988 | -0.0011 | 0.0002 | 4.14E-06 | 21.20 |
| rs13140136 | C | T | 0.1341 | 0.0009 | 0.0002 | 4.40E-06 | 21.08 |
| rs142165326 | G | A | 0.0131 | 0.0029 | 0.0006 | 1.95E-06 | 22.64 |
| rs150425398 | T | A | 0.0130 | 0.0031 | 0.0006 | 3.51E-07 | 25.95 |
| rs150531508 | A | G | 0.0102 | 0.0036 | 0.0007 | 5.39E-07 | 25.12 |
| rs2571227 | C | T | 0.3135 | 0.0007 | 0.0001 | 3.89E-06 | 21.32 |
| rs6900521 | G | A | 0.0122 | 0.0035 | 0.0006 | 3.04E-08 | 30.68 |
| rs7193065 | C | G | 0.6412 | 0.0007 | 0.0001 | 1.34E-06 | 23.37 |
| rs72686537 | C | A | 0.0100 | 0.0033 | 0.0007 | 4.42E-06 | 21.08 |
| rs72781767 | C | T | 0.0252 | 0.0020 | 0.0004 | 4.22E-06 | 21.16 |
| rs72839026 | T | G | 0.1826 | 0.0009 | 0.0002 | 1.59E-06 | 23.04 |
| rs75534956 | G | T | 0.0359 | 0.0019 | 0.0004 | 3.25E-07 | 26.10 |
| rs7589494 | T | C | 0.5147 | -0.0007 | 0.0001 | 1.50E-07 | 27.59 |
| rs78160590 | A | G | 0.0143 | 0.0031 | 0.0006 | 2.54E-07 | 26.57 |
| rs78420277 | A | G | 0.0179 | 0.0024 | 0.0005 | 4.19E-06 | 21.17 |
| rs78601792 | C | G | 0.2175 | -0.0008 | 0.0002 | 2.01E-06 | 22.59 |
| rs80090833 | C | T | 0.0223 | 0.0022 | 0.0005 | 2.59E-06 | 22.10 |
| rs9835089 | C | T | 0.0465 | -0.0015 | 0.0003 | 4.57E-06 | 21.01 |

**Table S12. 7 va****lid IVs used for MR analysis of ICH on major depressive disorder**

| SNP | Effect allele | Non-effect allele | Effect allele frequency | Beta | SE | *p* value | F Statistics |
| --- | --- | --- | --- | --- | --- | --- | --- |
| rs12318133 | G | A | 0.0988 | -0.0011 | 0.0002 | 4.14E-06 | 21.20 |
| rs13140136 | C | T | 0.1341 | 0.0009 | 0.0002 | 4.40E-06 | 21.08 |
| rs2571227 | C | T | 0.3135 | 0.0007 | 0.0001 | 3.89E-06 | 21.32 |
| rs7193065 | C | G | 0.6412 | 0.0007 | 0.0001 | 1.34E-06 | 23.37 |
| rs72839026 | T | G | 0.1826 | 0.0009 | 0.0002 | 1.59E-06 | 23.04 |
| rs7589494 | T | C | 0.5147 | -0.0007 | 0.0001 | 1.50E-07 | 27.59 |
| rs78601792 | C | G | 0.2175 | -0.0008 | 0.0002 | 2.01E-06 | 22.59 |

**Table S13. 7 val****id IVs used for MR analysis of ICH on attention deficit/hyperactivity disorder**

| SNP | Effect allele | Non-effect allele | Effect allele frequency | Beta | SE | *p* value | F Statistics |
| --- | --- | --- | --- | --- | --- | --- | --- |
| rs12318133 | G | A | 0.0988 | -0.0011 | 0.0002 | 4.14E-06 | 21.20 |
| rs13140136 | C | T | 0.1341 | 0.0009 | 0.0002 | 4.40E-06 | 21.08 |
| rs2571227 | C | T | 0.3135 | 0.0007 | 0.0001 | 3.89E-06 | 21.32 |
| rs72839026 | T | G | 0.1826 | 0.0009 | 0.0002 | 1.59E-06 | 23.37 |
| rs75534956 | G | T | 0.0359 | 0.0019 | 0.0004 | 3.25E-07 | 23.04 |
| rs7589494 | T | C | 0.5147 | -0.0007 | 0.0001 | 1.50E-07 | 26.10 |
| rs9835089 | C | T | 0.0465 | -0.0015 | 0.0003 | 4.57E-06 | 27.59 |

**Table S14. 19 va****lid IVs used for MR analysis of ICH on anxiety**

| SNP | Effect allele | Non-effect allele | Effect allele frequency | Beta | SE | *p* value | F Statistics |
| --- | --- | --- | --- | --- | --- | --- | --- |
| rs116535382 | G | C | 0.0124 | 0.0032 | 0.0007 | 9.55E-07 | 24.02 |
| rs12318133 | G | A | 0.0988 | -0.0011 | 0.0002 | 4.14E-06 | 21.20 |
| rs13140136 | C | T | 0.1341 | 0.0009 | 0.0002 | 4.40E-06 | 21.08 |
| rs142165326 | G | A | 0.0131 | 0.0029 | 0.0006 | 1.95E-06 | 22.64 |
| rs150425398 | T | A | 0.0130 | 0.0031 | 0.0006 | 3.51E-07 | 25.95 |
| rs150531508 | A | G | 0.0102 | 0.0036 | 0.0007 | 5.39E-07 | 25.12 |
| rs2571227 | C | T | 0.3135 | 0.0007 | 0.0001 | 3.89E-06 | 21.32 |
| rs6900521 | G | A | 0.0122 | 0.0035 | 0.0006 | 3.04E-08 | 30.68 |
| rs7193065 | C | G | 0.6412 | 0.0007 | 0.0001 | 1.34E-06 | 23.37 |
| rs72686537 | C | A | 0.0100 | 0.0033 | 0.0007 | 4.42E-06 | 21.08 |
| rs72781767 | C | T | 0.0252 | 0.0020 | 0.0004 | 4.22E-06 | 21.16 |
| rs72839026 | T | G | 0.1826 | 0.0009 | 0.0002 | 1.59E-06 | 23.04 |
| rs75534956 | G | T | 0.0359 | 0.0019 | 0.0004 | 3.25E-07 | 26.10 |
| rs7589494 | T | C | 0.5147 | -0.0007 | 0.0001 | 1.50E-07 | 27.59 |
| rs78160590 | A | G | 0.0143 | 0.0031 | 0.0006 | 2.54E-07 | 26.57 |
| rs78420277 | A | G | 0.0179 | 0.0024 | 0.0005 | 4.19E-06 | 21.17 |
| rs78601792 | C | G | 0.2175 | -0.0008 | 0.0002 | 2.01E-06 | 22.59 |
| rs80090833 | C | T | 0.0223 | 0.0022 | 0.0005 | 2.59E-06 | 22.10 |
| rs9835089 | C | T | 0.0465 | -0.0015 | 0.0003 | 4.57E-06 | 21.01 |

**Table S15. 17 vali****d IVs used for MR analysis of ICH on insomnia**

| SNP | Effect allele | Non-effect allele | Effect allele frequency | Beta | SE | *p* value | F Statistics |
| --- | --- | --- | --- | --- | --- | --- | --- |
| rs12318133 | G | A | 0.0988 | -0.0011 | 0.0002 | 4.14E-06 | 21.20 |
| rs13140136 | C | T | 0.1341 | 0.0009 | 0.0002 | 4.40E-06 | 21.08 |
| rs142165326 | G | A | 0.0131 | 0.0029 | 0.0006 | 1.95E-06 | 22.64 |
| rs150425398 | T | A | 0.0130 | 0.0031 | 0.0006 | 3.51E-07 | 25.95 |
| rs150531508 | A | G | 0.0102 | 0.0036 | 0.0007 | 5.39E-07 | 25.12 |
| rs2571227 | C | T | 0.3135 | 0.0007 | 0.0001 | 3.89E-06 | 21.32 |
| rs6900521 | G | A | 0.0122 | 0.0035 | 0.0006 | 3.04E-08 | 30.68 |
| rs7193065 | C | G | 0.6412 | 0.0007 | 0.0001 | 1.34E-06 | 23.37 |
| rs72686537 | C | A | 0.0100 | 0.0033 | 0.0007 | 4.42E-06 | 21.08 |
| rs72781767 | C | T | 0.0252 | 0.0020 | 0.0004 | 4.22E-06 | 21.16 |
| rs72839026 | T | G | 0.1826 | 0.0009 | 0.0002 | 1.59E-06 | 23.04 |
| rs75534956 | G | T | 0.0359 | 0.0019 | 0.0004 | 3.25E-07 | 26.10 |
| rs7589494 | T | C | 0.5147 | -0.0007 | 0.0001 | 1.50E-07 | 27.59 |
| rs78420277 | A | G | 0.0179 | 0.0024 | 0.0005 | 4.19E-06 | 21.17 |
| rs78601792 | C | G | 0.2175 | -0.0008 | 0.0002 | 2.01E-06 | 22.59 |
| rs80090833 | C | T | 0.0223 | 0.0022 | 0.0005 | 2.59E-06 | 22.10 |
| rs9835089 | C | T | 0.0465 | -0.0015 | 0.0003 | 4.57E-06 | 21.01 |

**Table S16. 18 valid IVs** **used for MR analysis of ICH on schizophrenia**

| SNP | Effect allele | Non-effect allele | Effect allele frequency | Beta | SE | *p* value | F Statistics |
| --- | --- | --- | --- | --- | --- | --- | --- |
| rs116535382 | G | C | 0.0124 | 0.0032 | 0.0007 | 9.55E-07 | 24.02 |
| rs12318133 | G | A | 0.0988 | -0.0011 | 0.0002 | 4.14E-06 | 21.20 |
| rs13140136 | C | T | 0.1341 | 0.0009 | 0.0002 | 4.40E-06 | 21.08 |
| rs142165326 | G | A | 0.0131 | 0.0029 | 0.0006 | 1.95E-06 | 22.64 |
| rs150425398 | T | A | 0.0130 | 0.0031 | 0.0006 | 3.51E-07 | 25.95 |
| rs150531508 | A | G | 0.0102 | 0.0036 | 0.0007 | 5.39E-07 | 25.12 |
| rs2571227 | C | T | 0.3135 | 0.0007 | 0.0001 | 3.89E-06 | 21.32 |
| rs6900521 | G | A | 0.0122 | 0.0035 | 0.0006 | 3.04E-08 | 30.68 |
| rs72686537 | C | A | 0.0100 | 0.0033 | 0.0007 | 4.42E-06 | 21.08 |
| rs72781767 | C | T | 0.0252 | 0.0020 | 0.0004 | 4.22E-06 | 21.16 |
| rs72839026 | T | G | 0.1826 | 0.0009 | 0.0002 | 1.59E-06 | 23.04 |
| rs75534956 | G | T | 0.0359 | 0.0019 | 0.0004 | 3.25E-07 | 26.10 |
| rs7589494 | T | C | 0.5147 | -0.0007 | 0.0001 | 1.50E-07 | 27.59 |
| rs78160590 | A | G | 0.0143 | 0.0031 | 0.0006 | 2.54E-07 | 26.57 |
| rs78420277 | A | G | 0.0179 | 0.0024 | 0.0005 | 4.19E-06 | 21.17 |
| rs78601792 | C | G | 0.2175 | -0.0008 | 0.0002 | 2.01E-06 | 22.59 |
| rs80090833 | C | T | 0.0223 | 0.0022 | 0.0005 | 2.59E-06 | 22.10 |
| rs9835089 | C | T | 0.0465 | -0.0015 | 0.0003 | 4.57E-06 | 21.01 |

**Table S17. 19 va****lid IVs used for MR analysis of ICH on neuroticism**

| SNP | Effect allele | Non-effect allele | Effect allele frequency | Beta | SE | *p* value | F Statistics |
| --- | --- | --- | --- | --- | --- | --- | --- |
| rs116535382 | G | C | 0.0124 | 0.0032 | 0.0007 | 9.55E-07 | 24.02 |
| rs12318133 | G | A | 0.0988 | -0.0011 | 0.0002 | 4.14E-06 | 21.20 |
| rs13140136 | C | T | 0.1341 | 0.0009 | 0.0002 | 4.40E-06 | 21.08 |
| rs142165326 | G | A | 0.0131 | 0.0029 | 0.0006 | 1.95E-06 | 22.64 |
| rs150425398 | T | A | 0.0130 | 0.0031 | 0.0006 | 3.51E-07 | 25.95 |
| rs150531508 | A | G | 0.0102 | 0.0036 | 0.0007 | 5.39E-07 | 25.12 |
| rs2571227 | C | T | 0.3135 | 0.0007 | 0.0001 | 3.89E-06 | 21.32 |
| rs6900521 | G | A | 0.0122 | 0.0035 | 0.0006 | 3.04E-08 | 30.68 |
| rs7193065 | C | G | 0.6412 | 0.0007 | 0.0001 | 1.34E-06 | 23.37 |
| rs72686537 | C | A | 0.0100 | 0.0033 | 0.0007 | 4.42E-06 | 21.08 |
| rs72781767 | C | T | 0.0252 | 0.0020 | 0.0004 | 4.22E-06 | 21.16 |
| rs72839026 | T | G | 0.1826 | 0.0009 | 0.0002 | 1.59E-06 | 23.04 |
| rs75534956 | G | T | 0.0359 | 0.0019 | 0.0004 | 3.25E-07 | 26.10 |
| rs7589494 | T | C | 0.5147 | -0.0007 | 0.0001 | 1.50E-07 | 27.59 |
| rs78160590 | A | G | 0.0143 | 0.0031 | 0.0006 | 2.54E-07 | 26.57 |
| rs78420277 | A | G | 0.0179 | 0.0024 | 0.0005 | 4.19E-06 | 21.17 |
| rs78601792 | C | G | 0.2175 | -0.0008 | 0.0002 | 2.01E-06 | 22.59 |
| rs80090833 | C | T | 0.0223 | 0.0022 | 0.0005 | 2.59E-06 | 22.10 |
| rs9835089 | C | T | 0.0465 | -0.0015 | 0.0003 | 4.57E-06 | 21.01 |

**Table S18. 18 va****lid IVs used for MR analysis of ICH on bipolar disorder**

| SNP | Effect allele | Non-effect allele | Effect allele frequency | Beta | SE | *p* value | F Statistics |
| --- | --- | --- | --- | --- | --- | --- | --- |
| rs116535382 | G | C | 0.0124 | 0.0032 | 0.0007 | 9.55E-07 | 24.02 |
| rs12318133 | G | A | 0.0988 | -0.0011 | 0.0002 | 4.14E-06 | 21.20 |
| rs13140136 | C | T | 0.1341 | 0.0009 | 0.0002 | 4.40E-06 | 21.08 |
| rs142165326 | G | A | 0.0131 | 0.0029 | 0.0006 | 1.95E-06 | 22.64 |
| rs150425398 | T | A | 0.0130 | 0.0031 | 0.0006 | 3.51E-07 | 25.95 |
| rs150531508 | A | G | 0.0102 | 0.0036 | 0.0007 | 5.39E-07 | 25.12 |
| rs2571227 | C | T | 0.3135 | 0.0007 | 0.0001 | 3.89E-06 | 21.32 |
| rs6900521 | G | A | 0.0122 | 0.0035 | 0.0006 | 3.04E-08 | 30.68 |
| rs72686537 | C | A | 0.0100 | 0.0033 | 0.0007 | 4.42E-06 | 21.08 |
| rs72781767 | C | T | 0.0252 | 0.0020 | 0.0004 | 4.22E-06 | 21.16 |
| rs72839026 | T | G | 0.1826 | 0.0009 | 0.0002 | 1.59E-06 | 23.04 |
| rs75534956 | G | T | 0.0359 | 0.0019 | 0.0004 | 3.25E-07 | 26.10 |
| rs7589494 | T | C | 0.5147 | -0.0007 | 0.0001 | 1.50E-07 | 27.59 |
| rs78160590 | A | G | 0.0143 | 0.0031 | 0.0006 | 2.54E-07 | 26.57 |
| rs78420277 | A | G | 0.0179 | 0.0024 | 0.0005 | 4.19E-06 | 21.17 |
| rs78601792 | C | G | 0.2175 | -0.0008 | 0.0002 | 2.01E-06 | 22.59 |
| rs80090833 | C | T | 0.0223 | 0.0022 | 0.0005 | 2.59E-06 | 22.10 |
| rs9835089 | C | T | 0.0465 | -0.0015 | 0.0003 | 4.57E-06 | 21.01 |
